# Adjusting for global effects in voxel-based morphometry: Gray matter decline in normal aging

**DOI:** 10.1016/j.neuroimage.2011.12.086

**Published:** 2012-04-02

**Authors:** Jonathan E. Peelle, Rhodri Cusack, Richard N.A. Henson

**Affiliations:** MRC Cognition and Brain Sciences Unit, Cambridge, UK

**Keywords:** Cognitive aging, Partial volume effects, Individual differences, Voxel-based morphometry

## Abstract

Results from studies that have examined age-related changes in gray matter based on structural MRI scans have not always been consistent. Reasons for this variability likely include small or unevenly-distributed samples, different methods for tissue class segmentation and spatial normalization, and the use of different statistical models. Particularly relevant to the latter is the method of adjusting for global (total) gray matter when making inferences about regionally-specific changes. In the current study, we use voxel-based morphometry (VBM) to explore the impact of these methodological choices in assessing age-related changes in gray matter volume in a sample of 420 adults evenly distributed between the ages of 18–77 years. At a broad level, we replicate previous findings, showing age-related gray matter decline in nearly all parts of the brain, with particularly rapid decline in inferior regions of frontal cortex (e.g., insula and left inferior frontal gyrus) and the central sulcus. Segmentation was improved by increasing the number of tissue classes and using less age-biased templates, and registration was improved by using a diffeomorphic flow-based algorithm (DARTEL) rather than a “constrained warp” approach. Importantly, different approaches to adjusting for global effects – not adjusting, Local Covariation, Global Scaling, and Local Scaling – significantly affected regionally-specific estimates of age-related decline, as demonstrated by ranking age effects across anatomical ROIs. Split-half cross-validation showed that, on average, Local Covariation explained a greater proportion of age-related variance across these ROIs than did Global Scaling. Nonetheless, the appropriate choice for global adjustment depends on one's assumptions and specific research questions. More generally, these results emphasize the importance of being explicit about the assumptions underlying key methodological choices made in VBM analyses and the inferences that follow.

## Graphical abstract

**Figure:**
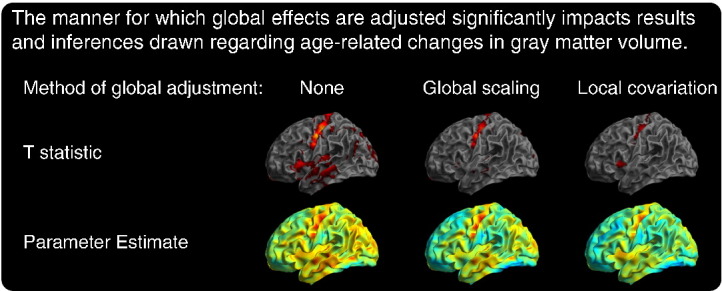

## Introduction

The presence of widespread decreases in gray matter (GM) in normal aging is well established, even in the absence of dementia or other neurological insult. Because these age-related changes vary across the brain, being able to accurately quantify regionally-specific effects over the lifespan has been of considerable interest for reasons that are both theoretical (e.g., identifying structure-function relationships by linking regional cortical change to behavior) and clinical (e.g., using regional cortical volume to aid in diagnosis of dementias).

Converging evidence for age-related GM loss comes from a variety of methodologies including brain weight measurements (Dekaban and Sadowsky, 1978, Miller et al., 1980, Peress et al., 1973), volumetric studies involving manual tracing of individual cortical regions (Allen et al., 2005, Raz et al., 1998, Raz et al., 2005), cortical thickness estimates based on surface models of the brain (Fjell et al., 2009, Salat et al., 2004), voxel-based cortical thickness measurements (Hutton et al., 2009), and voxel-based morphometry (VBM) (Good et al., 2001, Tisserand et al., 2004). Although there are advantages to each of these approaches, we have chosen to focus on VBM because it provides an objective measure of tissue volume, is widely used, and voxel-based statistics make it easier to integrate with other voxel-based analysis approaches such as used in fMRI or voxel-based cortical thickness (Das et al., 2009, Hutton et al., 2008).

At a basic level, the questions that researchers typically ask are “What areas of the brain show significant age-related change?” and “Which areas change more rapidly than others?”. Although widespread age-related decrease in GM has been found across a large number of studies, there is a lack of consensus about regional differences in age-related GM decline. In addition, there is a tendency (inherited from functional neuroimaging) for VBM studies to report only the significance level of age-related change in each voxel, rather than the magnitude of that change (i.e., effect size), which confounds answers to the two questions posed above. Beyond these theoretical points, there are several concrete methodological issues that contribute to a lack of agreement in VBM studies of aging. These include: (1) the use of relatively small and unevenly-distributed samples that tend to be skewed towards young adults; (2) differences in image processing (particularly in segmentation and registration); and (3) different statistical approaches (especially with respect to the treatment of global effects and the level of control exercised over false positives). To address these challenges, in the current study we used (1) a relatively large sample distributed evenly across adult ages (70 people per 6 decades from 18–77 years of age); (2) recent improvements in segmentation and registration methods implemented within the SPM8 software; and (3) a principled assessment of a range of approaches to adjusting for global effects, along with reporting of both statistics (with appropriate control of false positives) and effect sizes (Poldrack et al., 2008). Although it is common to express age-related change in relation to the “global” decrease in GM with age, we show that the results obtained, and the inferences permitted, depend on precisely how one adjusts for these global effects.

To illustrate the variability in the literature with respect to age-related GM change, and to focus on regions of interest (ROIs) for our present investigation, we selected a subset of studies using various methodologies to investigate age-related GM changes, listed in Table 1. For each study, we asked if there was age-related GM change in three regions: The middle frontal gyrus (MFG), chosen because of the interest in dorsolateral prefrontal cortex in “frontal” theories of age-related cognitive change; the central sulcus (CS), chosen because it is one of the most consistent regions to show age-related decline across both VBM and cortical-thickness studies; and the insula, chosen because it is one of the most consistent regions to show age-related decline in volumetric VBM studies but not in surface-based cortical-thickness studies. The lack of consensus regarding whether these three ROIs show significant age-related GM decrease is notable. (For brevity, we are unable to completely describe methodological differences between the studies cited in Table 1, which also include registration approach, smoothing kernel, statistical threshold, and whether the images are modulated to preserve the total amount of GM, any of which can also significantly influence the results.)

**Table 1:** Comparison of age-related gray matter decline in three regions of interest across selected studies.

	N	Age range	Software	TIV in model	TGM in model	MFG decline	CS decline	Insula decline
VBM								
Good et al. (2001)	465	18–79	SPM99	No	Yes	No^a^	Yes	Yes
Grieve et al. (2005)	223	8–79	SPM2	No	Yes	Yes	Yes	Yes
Lemaître et al. (2005)	662	63–75	SPM99	Yes^b^	No	Yes	Yes	No
Resnick et al. (2003)	92	59–85^c^	RAVENS	No	No	Yes	Yes	Yes
Taki et al. (2003)	769	16–79	SPM99	No	No	Yes	Yes	Yes
Tisserand et al. (2002)	57	21–81	SPM99	No	No	No	No	Yes
Tisserand et al. (2004)	75	> 50	custom	No	No	Yes	No	Yes
Present Study	420	18–77	SPM8	Yes	Yes/no^f^	No/yes	Yes/yes	Yes/yes
Cortical thickness								
Fjell et al. (2009)	883^d^	18–93	Freesurfer	No	No	Yes	Yes	No
Salat et al. (2004)	106	18–93	Freesurfer	No	No	No^e^	Yes	No
Sowell et al. (2003)	176	7–87	Custom	No	No	Yes	Yes	No

Note: TIV = total intracranial volume; TGM = total GMV; MFG = middle frontal gyrus; CS = central sulcus.

a There was MFG decline reported in the unmodulated analysis.

b Images were scaled by TIV, but it was not included as a voxelwise covariate.

c Longitudinal study with a five year follow up.

d Combined number of subjects across 6 studies.

e There was inferior prefrontal thinning that appeared to be inferior frontal gyrus, rather than MFG.

f This factor was varied in the present study, and the results changed.

A review of these studies highlights widespread variability in the way in which individual differences in brain size or total tissue class volume are statistically controlled. It is fairly straightforward to control for individual differences in overall head size by including total intracranial volume (TIV) as a covariate of no interest in the general linear model (GLM) that is fit to each voxel's data, although this is not always done.^2^ There is less consensus regarding how to handle global differences in a specific tissue class, like total GM (TGM: the sum of GM across all voxels), which is our primary consideration here. For example, when using VBM to compare young and older adults, the older group is likely to have less GM overall than the younger group. Assuming regional GM variations – rather than global differences – are of interest, one approach is to include each participant's TGM as an additional covariate of no interest in an Analysis of Covariance (ANCOVA), henceforth the “Local Covariation” approach. For each voxel, this effectively asks the question: To what extent is there a relationship between its GM and age that cannot be explained by the (linear) relationship between its GM and TGM? Because the parameter estimate for the linear effect of TGM is calculated separately for each voxel, the Local Covariation approach allows TGM to exert different effects in different voxels (resulting in different levels of adjustment at each voxel).

An alternative approach is to proportionally scale each participant's GM image by the TGM from that image, henceforth the “Global Scaling” approach. This asks the question: To what extent does GM in a voxel change with age at a rate over and above the rate of change of TGM with age? Global scaling enforces a consistent adjustment across voxels, and assumes that regionally-specific effects are proportional to, rather than additive with (as in Local Covariation), global effects like TGM.

A final approach to comparing age-related changes across regions is to scale the age effects by the mean GM across participants within each voxel (henceforth the “Local Scaling” approach). This asks the question: What is the rate of change of GM in a voxel, having adjusted for its mean GM? As we show in the Results, greater parameter estimates for the linear effect of age (i.e., a steeper slope) tend to be observed in voxels with greater overall local gray matter (LGM) (i.e., larger constant term in the GLM). It is conceivable that this trend reflects partial-volume effects or mis-segmentation; Local Scaling is thus one way to control for these possibilities.

**Fig. 1:**
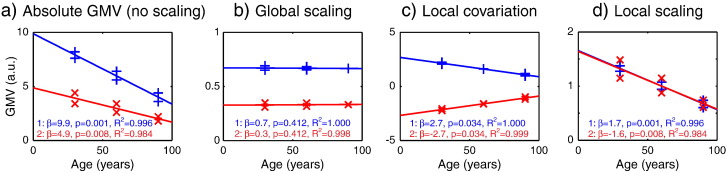
Hypothetical example of potential effects of different methods of adjusting for total GM (TGM). Data points reflect example GM volume (GMV, arbitrary units) for 2 regions in 6 participants, together with the best linear fit of age. The values in the bottom left of each graph are the parameter estimate for the slope (*β*_*1*_), the one-tailed p-value corresponding to that slope being different from zero, and the proportion of total variance (R^2^) explained by full model (linear and constant terms). (a) Unadjusted data (*y = β*_*1*_ *× age + β*_*2*_ *× Constant + Error*) show linear decline with different slopes for each region as a function of age. (b) A Global Scaling adjustment for TGM (*y/TGM = β*_*1*_ *× age + β*_*2*_ *× Constant + Error*) removes age effects, although the two regions still show different overall amounts of GM. (c) Local Covariation adjustment for TGM (*y = β*_*1*_ *× age + β*_*2*_ *× Constant + β*_*3*_ *× TGM + Error*) changes the slopes, with Region 2 now showing an increasing slope as a function of age. (d) A Local Scaling by an estimate of local GM (LGM) – the mean over participants for each region (*β*_*2*_) (*y = β*_*1*_ *× age + β*_*2*_ *× Constant + Error; β*_*1 =*_ *β*_*1*_ */ β*_*2*_*) –* results in nearly identical slopes for Region 1 and Region 2, both decreasing with age.

These different types of adjustment can dramatically alter the conclusions one makes about regional GM differences in aging. To illustrate this, consider a hypothetical example of GM estimated in two regions for six individuals (2 individuals at each of 30, 60 and 90 years of age), shown in Fig. 1. As typically found, we assume that GM decreases linearly with age in both regions, though twice as fast for Region 1 than for Region 2 (Fig. 1a). Fitting a linear regression model (a GLM including age and a constant term) to these data results in significantly negative linear slopes, which are numerically greater for Region 1 than Region 2, as expected. If TGM is used to scale the data for each participant – the Global Scaling approach (Fig. 1b) – then there are no longer any significant regionally-specific effects of age. Thus one would conclude that there is *no* significant evidence for age-related effects in either region, relative to (in a proportional sense) the global age-related decline. If TGM is instead included in the GLM with the unscaled data – i.e., the Local Covariation approach (Fig. 1c) – the age-related slope is again negative in Region 1, but becomes positive in Region 2, because the slope now represents changes relative to (in an additive sense) the average effect of age across the two regions. (Note also that the significance of the slopes is reduced relative to the case without scaling, while the overall model fit is increased, owing to removal of other individual differences in TGM.) Thus, one would conclude that there *are* regional differences in the effects of age (additive with global effects). Finally, consider scaling the data by the average GM for each region—the Local Scaling approach (Fig. 1d). Relative to the case in which no adjustment is made, Local Scaling does not affect the significance of the linear slopes (or overall model fit), but the size of the age-decline now differs little between the two regions; i.e., the two regions have roughly equivalent rates of GM change with age relative to their mean GM across individuals. Thus one would conclude that there *are* significant age-related effects in both regions, but these do not differ in size, relative to (in a proportional sense) their different mean GM values. These results clearly demonstrate how strikingly different conclusions might be drawn from identical data, depending on the type of adjustment made for TGM.

Having illustrated the different questions one might ask, and the potential for dramatically different answers depending on the precise form of global adjustment, below we consider how these various methods of adjustment affect the analysis of real data collected from a large sample of healthy adults.

## Method

### Participants

A total of 420 participants (214 male, 206 female) were included in this study. Participants were randomly selected from previous studies at the MRC Cognition and Brain Sciences Unit conducted between 2005 and 2010, constrained only to ensure equal representation across the age range in question (70 participants per decade from age 18–77 years). The majority were volunteers for functional MRI studies of language, attention, memory, etc., for which a structural T1-weighted image was also acquired. None were diagnosed with neurological difficulty at the time of scan and all were in good general health, assessed by self report and a standard pre-MRI screening questionnaire designed to highlight neurological difficulty. Structural scans of all participants (T1- and T2-weighted) were further reviewed by a neuroradiologist to ensure they were free of gross abnormalities. All participants completed an informed consent process approved by the local ethics review board.

### MRI acquisition

All images were acquired on the same Siemens 3 T Tim Trio scanner (Siemens Medical Systems, Erlangen, Germany) with a 12-channel head coil. During the period the scans were acquired there were no significant upgrades to the scanner hardware; only minor changes in software that Siemens state would not affect the sequences used here. We acquired a T1-weighted structural image for each participant using an MPRAGE sequence (TR = 2250 ms, TE = 2.99 ms, flip angle = 9°, FOV = 256 mm × 240 mm × 160 mm, voxel size = 1 mm × 1 mm × 1 mm).

### MRI segmentation

Prior to normalization using the diffeomorphic DARTEL approach, the MRI data were segmented into different tissue classes. Image processing was done using SPM8 release 4010 (Wellcome Trust Centre for Neuroimaging, London, UK) using the AA version 3.01 pipeline (http://www.cambridgeneuroimaging.com/aawiki/). Each individual's structural image was first coregistered to an ICBM152-space (i.e., MNI-space) average template distributed with SPM8 using normalized mutual information. This ensured reasonable starting estimates for the unified segmentation routine, and was done as an alternative to manually repositioning each scan. Structural images were then segmented into tissue classes using unified segmentation (Ashburner and Friston, 2005) as implemented in the “new segment” option of SPM8. This segmentation makes use of a number of tissue probability maps including GM, white matter (WM), cerebrospinal fluid (CSF), soft tissue, skull, and non-brain regions of the image. These maps reflect the prior probability of a given voxel belonging to a tissue class based on a large sample of healthy adults across the lifespan (Good et al., 2001). This information, in combination with the distribution of voxel intensities, is used to assign a probability to each voxel of belonging to a particular tissue class using Gaussian mixture modeling. Unless otherwise specified, default values were used for segmentation, except that the data were sampled every 1 mm (instead of the default 3 mm).

We also performed a second, parallel analysis using the alternative “standard” unified segmentation from SPM5/SPM8 (that has been used in many previous VBM studies). This segmentation uses fewer tissue classes (GM, WM, CSF), and tissue probability maps are based on a sample of young adults only (and hence potentially biased when examining age effects). Although not discussed at length in the main text, quite different effects of age were obtained from this “standard” segmentation analysis (shown in Supplemental Fig. S1–S3) relative to the “new” segmentation analysis reported in the main text.

Prior to segmentation, bias-corrected structural images were created to reduce the influence of intensity inhomogeneity on segmentation; producing a separate bias-corrected image effectively results in a two-pass bias correction, as bias correction is built-in to the segmentation process. Additionally, to reduce the likelihood that non-brain voxels were classified as GM, WM, or CSF, the tissue probability maps for these three tissue classes were set to 0 outside of a template-space brain mask.^3^ Following segmentation, images for each tissue class were roughly registered in a common space using a rigid body transformation. Segmented images were written out at 1.5 mm isotropic resolution.

The volume of the resulting GM, WM, and CSF tissue classes was determined from the (unsmoothed, unregistered) segmented images by integrating over all voxels and multiplying by voxel size, and the volumes of these three classes were summed to provide an estimate of total intracranial volume (TIV).

### MRI registration (spatial normalization)

The tissue class images created during segmentation were then used to generate a custom template using a diffeomorphic method known as DARTEL (Ashburner, 2007, Ashburner and Friston, 2009). This is achieved through an iterative process during which the parameters required to warp each subject into a common space are progressively refined. For each participant, flow fields were calculated during template creation that describe the transformation from each native GM image to the template; these were then applied to each participant's GM image. To transform these template-space images into ICBM152 space, the DARTEL template was registered to the tissue probability maps using an affine transformation, and this transformation then incorporated into the warping process. During this final normalization step, images were smoothed using an 8 mm FWHM isotropic Gaussian kernel.

As with the segmentation analyses above, we also performed a second parallel analysis using the “standard” registration/normalization in SPM5/SPM8. This normalization is based on a nonlinear warping to match a set of average tissue maps in ICBM152 space, expressed through a set of spatial basis functions (Ashburner and Friston, 2004). The warping is constrained by the spatial resolution of those basis functions, and regularized by minimizing the bending energy of the deformation fields. However, it is not a diffeomorphic flow-based method like DARTEL. Qualitatively, the resulting registration was less “sharp” than with DARTEL, and showed edge effects possibly related to poorer registration of older volunteers. Although not discussed at length in the main text, the results of this “constrained warp” analysis are shown in Supplemental Fig. S4.

When warping participants' images into a common space, an important decision concerns how to handle voxel intensities during the normalization process, given the spatial warping that occurs (Mechelli et al., 2005). One option is to leave the voxel intensities unchanged during normalization (“unmodulated” normalization). This preserves the concentration of GM in each voxel, but results in a change in the total amount of GM. A second option is to scale the intensity by the determinant of the Jacobian transformation matrix at each voxel—in other words, by the local amount of change in volume. This “modulated” analysis preserves the total amount of GM for each participant, and therefore provides a quantitative assessment of regional GM volume (GMV). For all analyses here, we used modulated images (i.e., volume preserved at each step from native space through to ICBM152 space).

All statistical analyses were explicitly masked using the same mask to ensure fair comparison (i.e., no threshold masking was used). For this purpose, a brain mask was created from the mean of all participants' smoothed DARTEL-normalized GM images, thresholded at 0.1 to create a binary mask.

### Statistical analysis

Our primary goal was to identify regions of GMV change associated with healthy aging. We therefore entered a second-degree polynomial expansion of age separately for each sex, enabling us to account for both linear and quadratic components of GMV change. We performed 12 separate analyses to investigate the effects of: (1) new versus standard segmentation in SPM8; (2) DARTEL versus standard registration in SPM8; and (3), most importantly, the three different methods of adjusting for TGM: no adjustment, Global Scaling and Local Covariation. We illustrate the equations corresponding to these latter effects below, using slightly simplified models for clarity.^4^

In the unadjusted analysis, GMV in a region (or a voxel) *r* for an individual *i* is modeled using their age *a*:

$$y_{\mathrm{ir}}=\beta_{r}^{\left(a\right)}a_{i}+\beta_{r}^{\left(c\right)}+\varepsilon_{\mathrm{ir}}$$

where *β*_*r*_ refers to the parameter estimate for either the effect of age (*a*) or the constant term (*c*) in this region, and *ε*_*ir*_ refers to the error in this region for this individual.

TGM for an individual, *t*_*i*_, is simply the sum of GM across regions, where $\tilde{y}$ refers to unregistered unsmoothed data:

$$t_{i}=\sum_{r}{\tilde{y}}_{\mathrm{ir}}$$

For the Global Scaling approach, each participant's smoothed normalized GM image was divided by the TGM for that participant:

$$\frac{y_{\mathrm{ir}}}{t_{i}}=\beta_{r}^{\left(a\right)}a_{i}+\beta_{r}^{\left(c\right)}+\varepsilon_{\mathrm{ir}}$$

For the Local Covariation approach, TGM for each subject was included in the GLM as a covariate:

$$y_{\mathrm{ir}}=\beta_{r}^{\left(a\right)}a_{i}+\beta_{r}^{\left(c\right)}+\beta_{r}^{\left(t\right)}t_{i}+\varepsilon_{\mathrm{ir}}$$

For the fourth type of adjustment – Local Scaling (which does not affect the statistics) – the statistical model is the same as in the unadjusted case above. However, the parameter estimate image for the linear slope effect (averaged across males and females) was divided by the parameter estimate image for the constant term in the model:

$$\beta_{r}^{\left(l\right)}=\frac{\beta_{r}^{\left(a\right)}}{\beta_{r}^{\left(c\right)}}$$

In the case where the age regressor *a*_*i*_ is mean-centered (as in the analyses reported here), we note that the parameter estimate for the constant term in the model is equivalent to the mean GM across all *n* participants, i.e., LGM:

$$\beta_{r}^{\left(c\right)}=\frac{\sum_{i}y_{\mathrm{ir}}}{n}$$

Finally, we included TIV as a covariate of no interest in every GLM. TIV significantly affects VBM results, and due to its non-proportional relationship with local GM changes is likely best accounted for using an ANCOVA approach (Barnes et al., 2010). This removes differences correlated with overall head size, given that our GM images were modulated by the degree of warping needed to register them to a template brain (otherwise the voxel values would include differences owing to overall head size). Nonetheless, note that the main conclusions regarding different methods of TGM adjustment remained in further analyses (not reported) in which no adjustment for TIV was made. (We also investigated the correlation between TIV and the other global measures, shown in Supplemental Fig. S3.)

For 3D surface rendering, template brains included in SPM8 were used. For slices, we displayed images on mean GM images using MRIcron (Rorden and Brett, 2000), available from http://www.mccauslandcenter.sc.edu/mricro/mricron/.

## Results

### Global measures of tissue volume

**Fig. 2:**
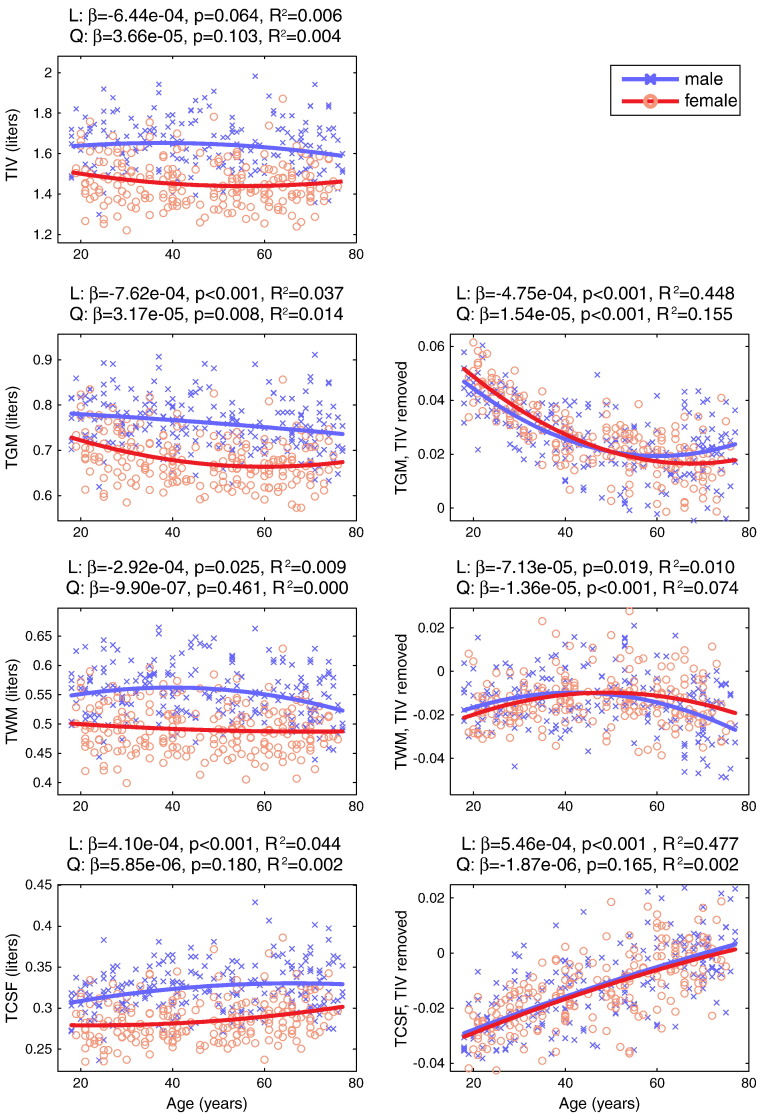
Plots of total intracranial volume (TIV) and total tissue class volume, both raw (left column) and after TIV has been factored out (right column), for the 420 male (blue) and female (red) participants. TGM = total gray matter, TWM = total white matter, TCSF = total cerebrospinal fluid. The parameter estimates (*β*), one-tailed p-values and partial R^2^ of for linear (L) and quadratic (Q) components are displayed above each plot, calculated across all participants (i.e., combined male and female).

Global measurements for TIV and total volume of the three major tissue classes (TGM, TWM, TCSF) are plotted as a function of age for males and females in Fig. 2, along with best-fit lines determined by a second-order polynomial expansion of age. Shown in the right column of Fig. 2 are the tissue class total volumes after TIV has been covaried out, again with best-fit lines. Before adjusting for TIV, there was approximately a 4% loss in TGM between 18 and 77 years of age (averaging across males and females), though age only explained approximately 5% of the total variance. After adjusting for TIV, there was a much larger and more negatively-accelerated decrease in TGM with age, with reliable linear and quadratic components that together explained approximately 60% of the remaining variance (and the difference between males and females was drastically reduced). TIV-adjusted TWM showed a small but significant linear decrease with age, which in combination with a quadratic effect explained 8% of the remaining variance. TIV-adjusted TCSF showed a larger linear increase with age that explained 47% of the remaining variance (with no reliable quadratic component).

The same graphs are shown in Supplemental Fig. S1 for the standard segmentation algorithm, and convey a somewhat different story. First, TIV decreased reliably with age, which was unexpected. Second, the decrease in (unadjusted) TGM with age was much greater, corresponding to approximately a 22% loss in TGM between 18 and 77 years of age. Thirdly, after TIV adjustment, TGM is decline was more linear (less negatively-accelerated). These results are more similar to those of Good et al. (2001), though note that in this previous report the authors scaled by TIV (a proportional adjustment), rather than adjusting for TIV (an additive adjustment) as we have done here. (We chose an additive adjustment to match the Local Covariation adjustment for TIV commonly performed in other voxelwise VBM models.) A direct comparison of the results for these two methods of segmentation is given in Supplemental Fig. S2. The differences apparent in this comparison highlight possible dangers of not using sufficient tissue classes (e.g., increasing the likelihood of a non-GM voxel being classified as GM) and/or using tissue probability maps that are biased towards younger adults.

With respect to accounting for global changes in whole-brain analyses, these data illustrate in a more concrete fashion two points noted previously. First, there is a significant decline in TGM as a function of age. This suggests that many regions of the brain will show significant age-related changes in GMV. Thus, simply looking for “significant change” in GMV without accounting for global changes is unlikely to be particularly informative, and studies of aging with small sample sizes that focus only on regions that pass a statistical threshold may be misleading (i.e., lack of significant age-related effects may simply be due to low power, given evidence that GMV in most parts of the brain decreases with age, discussed further below).

A second point suggested by examining these global measures is that TIV and TGM do not have identical relationships to age, suggesting that including TIV in the statistical model, although perhaps desirable for other reasons, will not necessarily control for TGM differences in this population. To examine this more quantitatively, we correlated these two global measures across participants, as shown in Supplemental Fig. S3. Although TGM and TIV showed reasonable dissociation in the standard segmentation approach, there was a considerable amount of shared variance between TIV and TGM in the new segmentation approach (a correlation value of .98). It is remarkable that, despite such a high correlation between these measures, it was still possible to identify unique effects of age after this variance has been accounted for (by inclusion of TIV in the GLM, as in Fig. 2.). The important implication of this is that the differences in TGM that could not be explained by TIV were systematic and strongly related to the aging process.

### Whole brain analyses

**Fig. 3:**
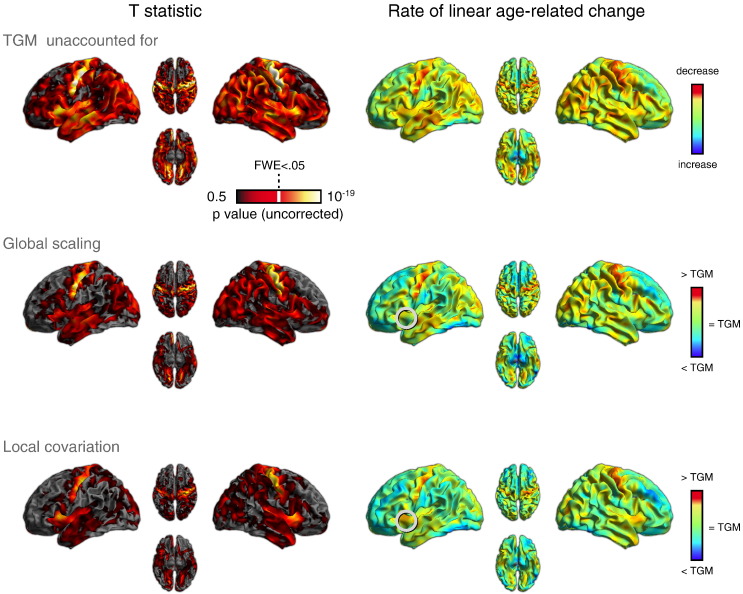
Linear age-related GMV declines for three different adjustment approaches for total GM (TGM): not adjusting for TGM, a Global Scaling approach, and a Local Covariation approach. Left column: one-tailed T statistic for the contrast of a negative linear effect of age, averaging over male and female participants. The white bar on the colorscale indicates voxelwise p_FWE_ < .05. Right column: parameter estimates from these same analyses. An example of differences between analysis approaches is highlighted by white circles (see text).

In a whole-brain analysis of age-related GMV change, we first looked for voxels showing a significant linear decrease with age (averaged across sex). Results from this analysis are shown in the left column of Fig. 3 for each of the different types of TGM adjustment (results for the quadratic effects are given Supplemental Fig. S5). Across adjustment type, age-related decline across the central sulcus was reliable; the same was true of age-related reductions in bilateral insulae. Age-related changes in MFG were more variable: Although small portions of MFG showed age-related decline in all analyses, most voxels above the inferior frontal sulcus no longer showed a reliable decline with age once adjusting for TGM. In general, adjusting for TGM, either by Global Scaling or Local Covariation, resulted in smaller regions of significant age-related change. However, the overall patterns of change were similar, which is reassuring given the theoretically possible divergences illustrated in Fig. 1 (although see Fig. 5 for differences).

Shown in the right-hand column of Fig. 3 are the parameter estimates for the linear decrease with age. These reflect the estimated rate of linear age-related change after factoring out the effect of the other regressors in the GLM. That is, when no global measures are taken into account (top row), the parameter estimates most nearly reflect the true rate of GMV change with advancing age (adjusting for TIV). When TGM is taken into account, the parameter estimates reflect the amount of age-related GM change after the effect of the global measure has been factored out. Thus, in the second row of Fig. 3 in which the Global Scaling approach is used, cool colors reflect an age-related change that is negative having adjusted for the global effect, and warm colors a positive age-related change after having adjusted for the global effect. In the Local Covariation approach, the interpretation is similar, except that the interpretation reflects the fact that the global effect has been covaried out at each voxel.

The effect of the different types of TGM adjustment on the voxelwise parameter estimates (Fig. 3, right column) was qualitatively similar to the effects on the statistics (Fig. 3, left column). That is, adjusting for TGM had a marked effect compared to not adjusting for TGM, but the differences between adjusting via Global Scaling compared with via Local Covariation were more subtle. However, divergences were apparent. For example, some voxels in left inferior frontal cortex (operculum) showed smaller age effects following Global Scaling than following Local Covariation, noted with white circles in Fig. 3. These cases illustrate the different conclusions that one might draw depending on the specific methods of TGM adjustment. Further differences in parameter estimates depending on the type of TGM adjustment are addressed in the ROI analysis below.

A comparison of age effects for DARTEL registration relative to the constrained warp registration is shown in Supplemental Fig. S4. At a relatively stringent voxelwise threshold of p_FWE_ < .05, the results of the DARTEL analysis appeared more focal than those from the constrained warp analysis. This is also seen in the mean GMV images shown in Fig. S4b, in which the DARTEL image appeared qualitatively crisper than the constrained warp image. Also evident from the slices is the tendency of the constrained warp approach to result in edge effects (indicated by white arrows) that were not apparent in the DARTEL analysis. These edge effects may reflect systematic registration difficulties in matching the (typically smaller) older adults' brains to a template weighted toward young adults.

### LGM scaling

**Fig. 4:**
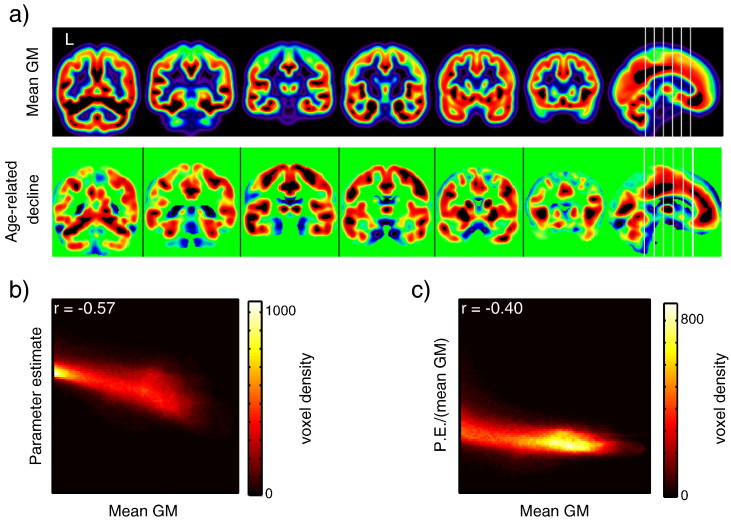
(a) Comparison of the mean GM image and the linear parameter estimate for the unadjusted TGM analysis shows qualitative similarities, with areas with large age-related change typically also having high mean GM values. (b) A quantitative comparison showing a scatter plot of all in-mask voxels. Voxels with higher mean GM values show more negative parameter estimates (indicating greater age-related decline). (c) When the mean GM is accounted for (i.e., the parameter estimate for the linear term is divided by that for the constant term), the strength of this relationship is reduced, but still significant.

In examining the regional variation in parameter estimates for linear age-related changes in GMV, we noticed what appeared to be a correspondence between larger age-related decreases and larger overall volumes of GM in a voxel, as shown in Fig. 4a (see also Supplemental Fig. S6). Specifically, both LGM and age effects appeared to be higher in the fundi of the cortical sulci, as might be expected (in the case of LGM) from partial volume effects. We quantified this dependence by plotting the parameter estimate for linear age-related decline against the parameter estimate for the constant term in the GLM on a voxel-by-voxel basis, shown in Fig. 4b (because the average error across all voxels in the estimate of this constant term was < 1%, this error was ignored). Due the large number of voxels included (~ 550,000), the density of voxels is shown using a color scale. There was indeed a significant negative correlation across voxels between age-related decline and LGM, Pearson r = − 0.57, p < .001. We then scaled the parameter estimate for the linear age effect by the LGM. The results of this scaling are shown in Fig. 4c. The dependence of the parameter estimate on LGM was reduced but still considerable, Pearson r = − 0.40, p < .001.

### Region of interest analysis

To complement the above whole-brain analysis of GMV change, we conducted a region of interest (ROI) analysis on the same data, and sorted the results by rate of linear GMV change. In other words, we asked the question: What regions of the brain showed the fastest age-related decline in GMV?

A total of 116 anatomical ROIs were defined using the AAL atlas (Tzourio-Mazoyer et al., 2002). For each region, we calculated the average linear effect size across all voxels using the same statistical model as in the whole-brain analyses, and then sorted the ROIs by decreasing average linear effect size for each type of global adjustment. We ensured normality of both the data and residuals in each region using Kolmogorov–Smirnov tests (p > .05 in all cases).

**Fig. 5:**
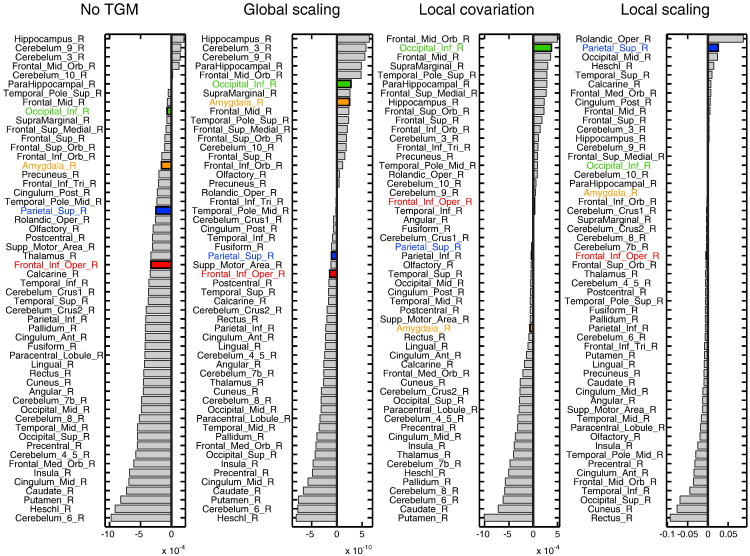
Analysis of the rate of linear GM change in 58 right hemisphere anatomical regions of interest (ROIs). For each of four adjustment approaches, ROIs are ranked by the average linear parameter estimate. Highlighted are four regions whose qualitative relationships change depending on the adjustment approach: inferior occipital cortex (green), amygdala (orange), superior parietal cortex (blue), and inferior frontal gyrus *pars opercularis* (red).

The results for the right hemisphere ROIs are shown in Fig. 5 (results from the left hemisphere are shown in Supplemental Fig. S7). It is notable, first, that in the unadjusted analysis nearly all ROIs showed a negative age effect (decreasing GMV with age). One general effect of global adjustment is to shift the division between positive and negative age effects, such that more ROIs now showed an increase of GMV with age (relative to global changes) when adjusted, as expected. For example, the inferior frontal gyrus *pars opercularis* ROI (highlighted in red) exhibited an age-related decline following adjustment by Global Scaling, but an age-related increase (albeit small) following adjustment by Local Covariation, confirming the impression from the (homologous) circled region in Fig. 3. Even more interesting is the fact that the different adjustments could also change the relative order of age effects across ROIs. For example, the inferior occipital ROI (highlighted in green) showed one of the highest rates of GMV increases relative to TGM following adjustment by Local Covariation, but not after Local Scaling. Or as a further example, the conclusion one would draw about the relative effect of age on the amygdala (orange) versus superior parietal cortex (blue) would reverse depending on the analysis: the parietal ROI had a more negative slope than the amygdala ROI in the analysis without TGM adjustment and in the analysis using Global Scaling (leftmost two panels of Fig. 5), but the amygdala ROI had a more negative slope than the parietal ROI in the analyses using Local Covariation or Local Scaling (rightmost two panels).

**Fig. 6:**
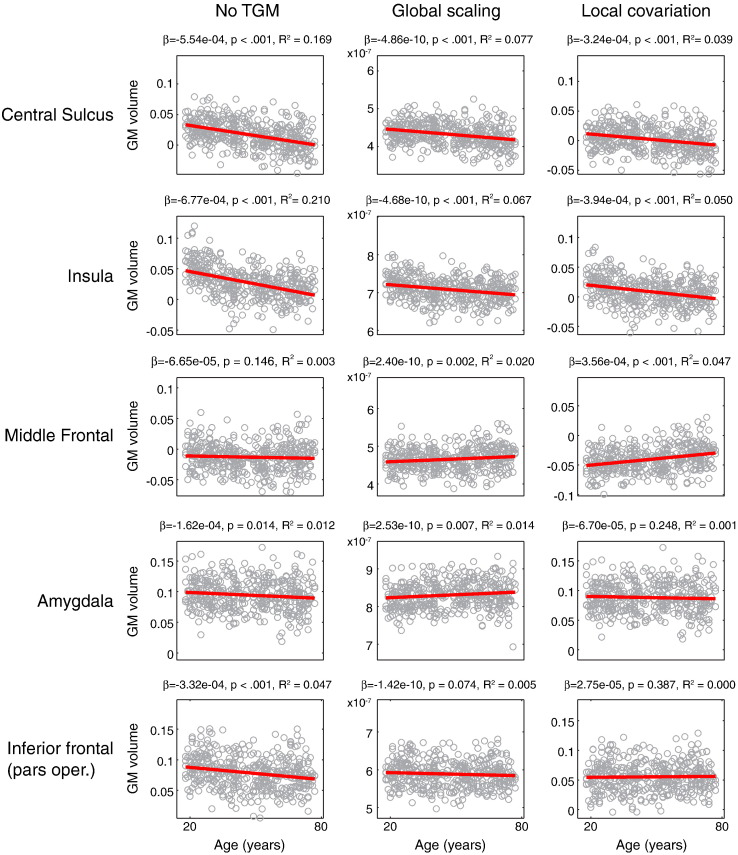
For five right-hemisphere regions of interest (ROIs), GM values averaged across voxels within each ROI, after correcting for TIV, are shown, along with a best linear fit. Each column reflects a different adjustment for total GM (TGM) in the statistical model: not controlling for TGM, a Global Scaling approach, and a Local Covariation approach. The parameter estimates, one-tailed p-values and partial R^2^ (linear component of age effect) are displayed above each plot.

Fig. 6 shows GMV as a function of age for five example ROIs, along with a best-fitting linear regression line, for each of the three TGM adjustment approaches (Local Scaling is not shown, as this does not affect the statistics of the age effects). These five regions included our three a priori ROIs, plus two regions (amygdala and inferior frontal operculum) whose relative rank depended on the type of TGM adjustment in Fig. 5. Although the statistics shown are from the full model, which includes TIV, for display purposes we have plotted the residuals after TIV removal (which also matches our previous analyses). There are two primary points to note in these plots. First, the overall amount of age-related change was relatively small in relation to the individual differences (explaining at most 21% of the total variance, in the case of the insula without TGM adjustment). Second, the nature of the relationship between GM and age (i.e., the slope of the regression line) changed with the type of TGM adjustment (again, even changing in sign for some ROIs, such as middle frontal gyrus).

### Generalization of Local Covariation vs. Global Scaling adjustments

As noted previously, various approaches to controlling for TGM are probably best viewed as testing different hypotheses, rather than competing tests of the same hypothesis. Nonetheless, it may still be productive to examine which of the approaches we consider here “best” fit the data, particularly in terms of cross-validation (generalization from one half of the sample to the other half). We did this by examining the amount of age-related variance explained by the Global Scaling and Local Covariation models across the 116 anatomical ROIs used above.

**Fig. 7:**
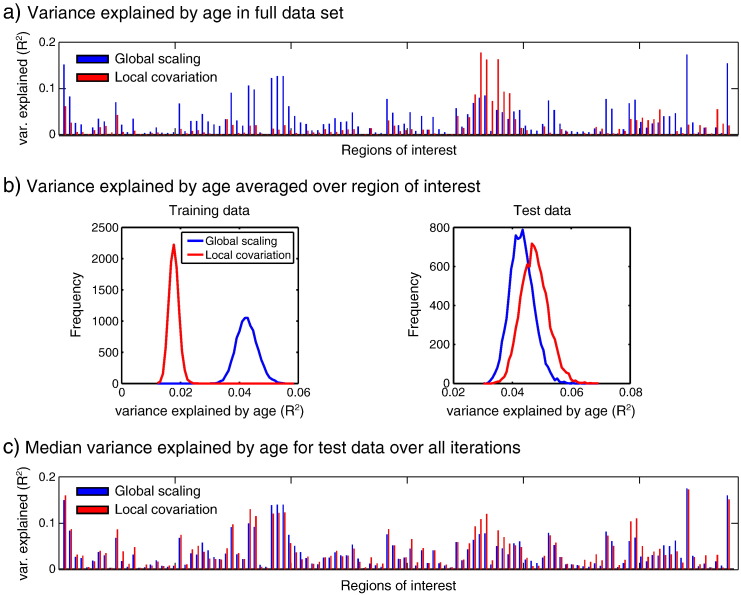
Comparison of TGM adjustment methods in terms of variance explained by age. (a) Variance explained by age in the full data set after adjusting for TGM using Global Scaling or Local Covariation for each of 116 anatomical ROIs. (b) Variance explained by age across 10,000 split-half cross-validations for training and test sets, averaged over ROI. (c) Median variance explained by age across iterations in the test data based on estimates of age-related change in the training data, plotted by ROI (note that the difference in minimum/maximum values across panels b and c reflects the summarizing over ROIs or over iterations, respectively, with the latter having a more skewed distribution).

First, for each ROI, we fit a separate GLM for each method of TGM adjustment. For Global Scaling, the model included a second-order polynomial expansion of age and TIV, and was fit to the scaled data. For Local Covariation, the TGM regressor was added to the GLM, and fit to the unscaled data. In both cases, we report the R^2^ value reflecting the proportion of variance explained by combined linear and quadratic age effects. These values are shown for each ROI in Fig. 7a, which show that Global Scaling explained more age-related variance than Local Covariation in 92/116 regions.

We next tested generalizability using a cross-validation approach. For each of 10,000 iterations, we randomly selected half of the participants with which to fit each GLM. We refer to this half as the training data, with the remaining participants being the test data. For each iteration, we used the parameter estimates for the linear and quadratic age effects resulting from fitting the training data, and calculated the proportion of variance that these estimates explained in the test data. For Global Scaling, the test data were scaled by their TGM (not the TGM of the training data):

$$\frac{GMV_{\mathrm{test}}}{TGM_{\mathrm{test}}}=X_{\mathrm{test}}\times \beta_{\mathrm{train}}+Error$$

where *X*_*test*_ is the design matrix containing linear and quadratic age terms, and *β*_*train*_ is a vector of the corresponding parameter estimates. Similarly, for Local Covariation, the test data were adjusted by subtracting the fit of the test TGM to the test data:

$$GMV_{\mathrm{test}}-\beta TGM_{\mathrm{test}}\times TGM_{\mathrm{test}}=X_{\mathrm{test}}\times \beta_{\mathrm{train}}+Error$$

Fig. 7b shows a histogram across iterations of the mean proportion of remaining variance explained by age, averaged over ROIs, for both the training and test data sets. In the training data, age explained more variance after Global Scaling (as in Fig. 7a). However, in the test data, age explained more variance after Local Covariation. This is also apparent when median variance explained in the test data across iterations is plotted by ROI, shown in Fig. 7c, in which age explained more variance after Local Covariation in 73/116 regions. These findings suggest that the age-related change estimated after Local Covariation will generalize better to a new sample of participants than that estimated using a Global Scaling adjustment for TGM.

## Discussion

In this report we investigated age-related change in GMV in 420 adults aged 18–77. We were interested in exploring the effects of different preprocessing and analysis approaches, and how these results could inform our view of the normally aging brain. Below we consider points related to each of these topics in turn.

### Effects of tissue class segmentation

Although differing approaches to tissue class segmentation were not the primary focus of this report, we briefly compared the “new” segmentation approach in SPM8 with the “standard” SPM5/SPM8 unified segmentation approach. Qualitative differences were seen in the global levels of tissue classified, the effect of age on these global values, and correlations between various global measures (cf. Fig. 1, Fig. 2, Fig. 3 and S1–S3). These differences highlight the importance of segmentation approach on all subsequent stages of analysis. In the current context, they also emphasize the point that, to the extent that global data properties differ across segmentation approaches, controlling for these global effects will result in different inferences (for example, with respect to differential regional age-related changes).

There are at least two possible explanations for the differences across segmentation approach. First, the new segmentation approach makes use of a larger number of tissue probability maps, and in theory should thus be more accurate at assigning voxels to the appropriate tissue class. Second, the tissue probability maps from the new segmentation approach were derived from a sample of adults ranging in age from approximately 18–79 (J. Ashburner, personal communication), compared to the ICBM152-based maps used in the standard segmentation that were based solely on a sample of younger adults. The age composition of these probability maps could cause artifactual age effects in the segmentation process. An important goal for future studies is to continue to assess how the choice of tissue probability maps and segmentation process influence segmentation, particularly when participants are not healthy young adults (Acosta-Cabronero et al., 2008, Wilke et al., 2008).

### Effects of registration approach

Although several key aspects of our results replicated across registration approach, there were also noticeable differences. Results obtained using DARTEL were generally more focal than those obtained using the constrained warp registration to an a priori template (i.e., spatial normalization). This observation is consistent with previous evaluations showing greater overlap across subjects of macroanatomical cortical areas for high-dimensional registration approaches (Klein et al., 2009). There were also some regions, including bilateral insulae, that more consistently exhibited significant age-related GMV change in the DARTEL-based analysis, which may reflect better alignment of these structures across participants. Again, there are at least two possible explanations for the differences in these registration approaches: first there is the nature of the registration algorithm itself (i.e., based on flow fields or spatial basis functions) and second there is the distinction between a template specific to the sample (as in DARTEL) and a template based on a different sample (as in the constrained warp approach, which uses a template that may be biased towards younger brains). Indeed, as noted above, the “edge effects” in the constrained warp analysis may have arisen from an inability of the standard normalization approach to warp the (relatively smaller) older adult brains in the present sample to the a priori template, resulting in systematic decreases in GMV around the edge of the brain.

### Common effects of age-related gray matter atrophy

Despite the above differences as a function of preprocessing choices, there were patterns of age-related change identified in the whole-brain analysis that were qualitatively similar across analysis approach (including across the methods of adjusting for TGM). Most obvious among these was significant age-related decrease in GMV along the central sulcus bilaterally, as well as in the insulae. These effects were evident independent of whether TGM was adjusted for. However, as noted, even for regions that consistently show significant decline, the parameter estimates can differ markedly (recall the inferior frontal operculum in Fig. 3 and its different rankings in Fig. 5).

In relating our findings to previous studies, comparison across methodologies is potentially interesting, particularly with regard to the age-related change that we observed along the central sulcus. VBM studies are divided in those that do (Good et al., 2001, Grieve et al., 2005, Resnick et al., 2003) or do not (Tisserand et al., 2002, Tisserand et al., 2004) report significant atrophy along the central sulcus. However, surface-based methods appear slightly more consistent in this regard: Both Fjell et al. (2009) and Salat et al. (2004) report significant thinning in this region using surface-based cortical thickness measures (although this trend is less apparent in Sowell et al., 2003). Perhaps most relevant investigation is a study by Hutton et al. (2009) in which VBM and voxel-based cortical thickness (VBCT) measurements were compared in the same group of participants. Qualitative comparison of parameter estimates across these analyses suggest that VBM indicated a greater decrease in more anterior portions of frontal cortex (i.e., large areas of dorsolateral prefrontal cortex, including inferior, middle, and superior frontal gyri), whereas VBCT showed relatively less decrease in this regions, but large changes along the central sulcus. The differences in detecting this change across VBM studies is unlikely to be due solely to changes in registration approach, as we observed GM declines in this region regardless of whether normalization was done via DARTEL or constrained warping. Similarly, these differences across VBM studies are unlikely to be due to different treatment of TGM, because we observed decreases in the central sulcus consistently across analysis type. On balance therefore it seems that the majority of the evidence points to central sulcus thinning being a reliable consequence of normal aging, although the basis for the remaining inconsistencies remains to be resolved.

The age-related decrease in insular GMV is also a finding that shows considerable heterogeneity across studies and methodology: surface-based methods do not tend to report insular atrophy (Fjell et al., 2009, Salat et al., 2004), although VBCT does (Hutton et al., 2009), and VBM studies consistently report age-related decreases in these regions (Good et al., 2001, Grieve et al., 2005, Resnick et al., 2003, Tisserand et al., 2004). In this case, the more striking differences between surface- and volume-based methods may suggest something intrinsic about the registration of the insula. In fact, the overall lower variability in regions that tend to show the strongest age effects (Supplemental Fig. S6b) and concomitant lower model error (Supplemental Fig. S6c) suggest there may be significant effects of registration accuracy.

### Effects of global covariates

For the remainder of the discussion, we focus on the results from the new segmentation and DARTEL analysis, and consider differences as a function of the type of adjustment for TGM. Globally scaling each participant's GMV image by their TGM resulted in a higher proportion of the remaining variance being age-related in most anatomical ROIs, compared to subtracting TGM-related variance in the Local Covariation approach. However, when we compared the ability of age-related effects estimated from one half of the data to predict age-related effects in the other half, we found better generalization for the Local Covariation approach. Because we do not know the true extent of age-related variance in TGM-adjusted data, this does not mean that the Local Covariation is necessarily a superior method of TGM adjustment, but it might be a reason to use Local Covariation in the absence of other information (i.e., lacking a specific hypothesis about the relationship between local GMV, TGM and age). Nonetheless, it must be remembered that different methods for adjusting for TGM are in fact testing different hypotheses. Thus, in the case of the central sulcus, consistency across analysis type does not add support for a single conclusion (e.g., “Gray matter volume in the central sulcus declines with age”), but rather provides evidence for three separate conclusions:

1. GMV in the central sulcus declines significantly with age (TGM not accounted for);
2. GMV in the central sulcus declines significantly with age at a rate greater than TGM change with age (Global Scaling);
3. GMV in the central sulcus declines significantly with age despite any changes in that region that are linearly related to TGM (Local Covariation).

In choosing an appropriate analysis approach, it is worth considering the theoretical framework within which to view brain–behavior relationships. That is, is it the absolute amount of cortical matter present that is of interest, or how that amount compares to GMV elsewhere in the brain? These questions are not mutually exclusive, and it could well be that there are cases for which more than one analysis could be conducted.

In addition to some brain areas in which results are superficially similar, there are also clear differences across analysis. The middle frontal gyrus for example, though difficult to define anatomically, showed evidence of absolute age-related decline, but not a decline that was significantly greater than the global TGM decline with age (Fig. 3, Fig. 6); i.e., would be associated with Conclusion 1 above, but not Conclusions 2–3. This may explain some of the discrepancies concerning MFG between the studies listed in Table 1. As well as inferences about single regions, the relative size of age effects across regions is also markedly affected by the method of TGM/LGM adjustment. This is perhaps best illustrated by the comparison of ROI rankings in Fig. 5, in which the relative age-related change across many ROIs differed across analyses. Given that there are cases in which these different analysis approaches would not agree, inferences made need to be sufficiently specific (e.g., whether age-related GMV changes are best expressed in absolute terms, or after adjusting in an additive or proportional manner for TIV, TGM and/or LGM).

In summary, there are two complementary ways of thinking about the differences across analysis approach. Perhaps the most obvious is that different ways of controlling for TGM will give different “results” (i.e., parameter estimates are not identical). However, even the same numerical result will have a different interpretation based on what else is included in the statistical model. These points also emphasize the importance of considering effect sizes (and what they represent), and not just statistics, in interpreting analyses.

### Effects of Local Scaling

In the course of examining our results, we noted a qualitative correspondence between the estimated mean GMV in a voxel, across all participants, and the estimated size of (linear) age-related change. This observation was supported by a statistically significant relationship between these two measurements, such that voxels with larger LGM values (larger parameter estimate for the constant term in the GLM) tended to show greater slopes of age-related decline. This relationship persisted even when controlling for LGM by scaling the parameter estimate for the linear effect by that for the constant term in the GLM, although the correlation coefficient was reduced. The question, then, is whether the relationship between mean GMV and estimates of age-related change might reflect something interesting about cortical organization and aging, or whether it is simply an artifact of image processing.

One possible explanation is that this relationship is driven, at least in part, by partial volume effects. That is, voxels that contain a larger proportion of GM across participants (and thus a higher mean GMV value) provide a more precise estimate of age-related change. A second, more speculative, possibility is that there is a relationship between neurotrophic and aging processes in the brain, such that regions with greater GMV show more age-related vulnerability (at least in those areas in which partial volume effects are minimal, such as sulcal fundi). This is particularly intriguing given that the depths of major sulci – sulcal pits – materialize early in development (Rakic, 1988), appear to be strongly genetically influenced (Piao et al., 2004), and are relatively well preserved across individuals (Im et al., 2010, Lohmann et al., 2008).

In some ways, the current situation echoes that previously faced by researchers in functional neuroimaging studies (Markowitsch and Tulving, 1994), and more generally highlights the question of the circumstances under which regional variation in neuroimaging measures can be attributed to neurobiology rather than methodology. If partial volume effects are indeed responsible, it may be desirable to correct for these effects in VBM studies. Simple ways to control for the influence of LGM on the results include the simple approach as we have implemented here (Local Scaling). However, more complex methods of adjusting for partial volume effects may also prove useful. Further studies are needed to better characterize the reasons for this relationship, and the most sensible ways to take it into account when analyzing data.

In one relevant study, Taki et al. (2011) examined the dependency on age-related GMV change and baseline GMV values in a 6-year longitudinal VBM study, restricted to ROIs that showed significant age-related volume decrease. Unlike the present analysis, they found that participants with lower levels of initial GMV in hippocampus and precuneus showed larger amounts of age-related GMV decreases. In the context of such a focused longitudinal study, this negative relationship may be linked to individual differences in the aging process (or early signs of neuropathology). Thus, the relationship between LGM and age-related change seems likely to differ depending on the specific population being investigated.

### Implications for theories of cognitive aging

To the extent that cognitive functions are supported by circumscribed regions of the brain, the heterogeneity in GMV decline reported here and elsewhere suggests that cognitive functions should show a corresponding variability in their pattern of preservation and decline. Alternatively, if these corresponding cognitive declines are not observed, it might suggest that older adults are able to compensate for the consequences of neurobiological change, and maintain a consistent level of behavior. These options are not mutually exclusive, and in fact, there is ample support for both in the literature.

Although there is certainly interest in accounting for the myriad of age-related changes in cognition using a small number of variables (Lindenberger and Baltes, 1994, Salthouse, 1996), it is clear that there is a great deal of variability older adults' behavior across cognitive domains. For example, relatively substantial age-related declines are consistently reported in episodic memory tasks (Craik, 1977; Golomb et al., 2008, Naveh-Benjamin, 2000, Wingfield et al., 1998), whereas implicit memory is generally less affected (Light et al., 2000, Rybash, 1996). Likewise, not all language processes are impacted in aging: older adults are consistently differentially impaired in the face of acoustic degradation (Gordon-Salant and Fitzgibbons, 2001, Letowski and Poch, 1996, Peelle and Wingfield, 2005), competing speech or noise (Tun and Wingfield, 1999, Tun et al., 2002), and some types of word production (Burke et al., 1991, Shafto et al., 2007). At the same time, online syntactic processing is generally found to be relatively well preserved (Fallon et al., 2006, Tyler et al., 2010, Waters and Caplan, 2001). These dissociations in behavioral performance are consistent with anatomical heterogeneity of neurobiological change.

The ability to maintain stable behavior despite wide variability in underlying physiology is a fundamental property of biological systems (Prinz et al., 2004). In keeping with this view, a ubiquitous theme in the cognitive aging literature has been one of compensation—that is, mechanisms by which older adults recruit additional cognitive (neural) resources to overcome deficits in primary cognitive (neural) systems. This is supported by the observation that across a wide variety of tasks, older adults often make less use of the (presumably) specialized networks used by young adults (e.g., Cabeza et al., 2002, Duarte et al., 2008, Gutchess et al., 2005, McIntosh et al., 1999, Park et al., 2004, Peelle et al., 2010, Tyler et al., 2010). To the degree that recruitment of additional brain regions supports successful performance, it can be viewed as compensatory (Cabeza, 2002, Wingfield and Grossman, 2006). However, there are other tasks in which older adults show essentially the same pattern of recruitment as young adults (Lustig and Buckner, 2004); again, these dissociations in the degree and nature of neural recruitment are consistent with heterogeneity of GMV loss reported here and elsewhere.

With regard to specific patterns of age-related change, the distribution of frontal atrophy observed in the current study deserves special mention. The selective vulnerability of frontal and prefrontal cortices has long been known (Brody, 1955, Haug, 1987, Raz et al., 1997) and ingrained into the cognitive aging literature (Dempster, 1992, West, 1996), although consensus has not been reached regarding the behavioral consequences of this decline (Greenwood, 2000, West, 2000). In the current study we found significant frontal atrophy, but it was confined largely to regions bordering the central sulcus and the insula, extending only slightly into inferior frontal gyrus. Notably, large portions of inferior, medial, and superior frontal gyri – including canonical “dorsolateral prefrontal cortex” – did not show significant decline in the voxelwise whole-brain analysis. However, we also noted that these regions showed increased variability (Fig. S6b), and thus one possibility is that registration difficulties prevented the detection of true age-related change. This interpretation is consistent with the clear effect of age on frontal GMV in our ROI analysis, as well as the more extensive detection of decreases in prefrontal volume in manual tracing studies (Raz et al., 1997) and surface-based approaches (Fjell et al., 2009, Salat et al., 2004). Clearly, further work is needed to better reconcile these findings, and thus set the stage for more accurate assessment of brain–behavior relationships.

Another point worth emphasizing is the relatively small contribution of age-related change relative to the local variability in GMV across participants (even after adjusting for TIV), as evident from the plots from individual ROIs in Fig. 6. This observation suggests that, to the extent that absolute cortical volume is related to cognitive function, aging per se may play a somewhat minimal role, merely modifying existing differences in neuroanatomy that reflect a combination of genetic factors and environmental influences. The relationship of age-related change to overall individual variability is important in interpreting correlations of GMV and behavioral and/or other neural measures.

Finally, with respect to VBM analyses, we have highlighted the importance of interpreting regional GMV change in relation to other covariates in the model. This same principle also applies to interpreting brain–behavior relationships. That is, for example, a lack of correlation between TGM-scaled GMV and a behavioral variable does not mean there is not a significant relationship between GMV and the behavior; it means that there is not a significant relationship above and beyond that which can be explained by global changes.

## Conclusions

Automated methods for analyzing GM have done much to advance our understanding of brain structure and how it changes during the course of development, normal aging, and disease. With respect to normal aging, we broadly replicate what numerous studies have consistently demonstrated: age-related declines in GM are not evenly distributed over the brain, but show a regional specificity. In our study, the regions of GMV loss were most pronounced along the central sulcus and in the insulae, as well as along the left middle and superior temporal gyri. However, it is clear that methodological considerations can significantly influence the results obtained and the inferences that can be drawn. Here we show, specifically, how the treatment of TGM in the statistical model can influence estimates of age-related change. We also demonstrate notable effects of segmentation approach and registration algorithm, highlighting the dependence of VBM results on these methodological choices. Finally, we show that LGM (i.e., the mean GM for a region across participants) is significantly related to the observed magnitude of age-related decline. Together, these findings underscore that careful attention to differences in approach, and being explicit about inferences being drawn, continue to be essential in interpreting VBM results.

## Footnotes

2 This may not be necessary if the voxel values have not been modulated by the affine part of the warping parameters, which capture differences in head size (Buckner et al., 2004).

3 The segmentation routine uses the logs of the tissue probability maps, and thus values of 0 are problematic. However, SPM adds a small value before computing the log values, which ensures that these values are not encountered.

4 To facilitate comparison, these simplified models do not include a term for TIV, and include only a single parameter for the age effect, rather than separating linear and quadratic components for both males and females as done in our actual analyses.

## References

[bb0005] Acosta-Cabronero J., Williams G.B., Pereira J.M.S. (2008). The impact of skull-stripping and radio-frequency bias correction on grey-matter segmentation for voxel-based morphometry. NeuroImage.

[bb0010] Allen J.S., Bruss J., Brown C.K., Damasio H. (2005). Normal neuroanatomical variation due to age: the major lobes and a parcellation of the temporal region. Neurobiol. Aging.

[bb0020] Ashburner J. (2007). A fast diffeomorphic image registration algorithm. NeuroImage.

[bb0500] Ashburner J., Friston K.J., Frackowiak R.S.J., Friston K.J. (2004). Spatial normalisation using basis functions. Human Brain Function.

[bb0015] Ashburner J., Friston K.J. (2005). Unified segmentation. NeuroImage.

[bb0025] Ashburner J., Friston K.J. (2009). Computing average shaped tissue probability templates. NeuroImage.

[bb0030] Barnes J., Ridgway G.R., Bartlett J., Henley S.M.D., Lehmann M., Hobbs N., Clarkson M.J., MacManus D.G., Ourselin S., Fox N.C. (2010). Head size, age and gender adjustment in MRI studies: a necessary nuisance?. NeuroImage.

[bb0035] Brody H. (1955). Organization of the cerebral cortex. III. A study of aging in the human cerebral cortex. J. Comp. Neurol..

[bb0040] Buckner R.L., Head D., Parker J., Fotenos A.F., Marcus D., Morris J.C., Snyder A.Z. (2004). A unified approach for morphometric and functional data analysis in young, old, and demented adults using automated atlas-based head size normalization: reliability and validation against manual measurement of total intracranial volume. NeuroImage.

[bb0045] Burke D.M., MacKay D.G., Worthley J.S., Wade E. (1991). On the tip of the tongue: what causes word finding failures in young and older adults?. J. Mem. Lang..

[bb0050] Cabeza R. (2002). Hemispheric asymmetry reduction in older adults.

[bb0055] Cabeza R., Anderson N.D., Locantore J.K., McIntosh A.R. (2002). Aging gracefully: compensatory brain activity in high-performing older adults. NeuroImage.

[bb0505] Craik F.I.M., Birren J.E., Schaie K.W. (1977). Age differences in human memory. Handbook of the Psychology of Aging.

[bb0060] Das S.R., Avants B.B., Grossman M., Gee J.C. (2009). Registration based cortical thickness measurement. NeuroImage.

[bb0065] Dekaban A.S., Sadowsky D. (1978). Changes in brain weights during the span of human life: relation of brain weights to body heights and body weights. Ann. Neurol..

[bb0070] Dempster F.N. (1992). The rise and fall of the inhibitory mechanism: toward a unified theory of cognitive development and aging. Dev. Rev..

[bb0075] Duarte A., Henson R.N., Graham K.S. (2008). The effects of aging on the neural correlates of subjective and objective recollection. Cereb. Cortex.

[bb0080] Fallon M., Peelle J.E., Wingfield A. (2006). Spoken sentence processing in young and older adults modulated by task demands: evidence from self-paced listening. J. Gerontol. B. Psychol..

[bb0085] Fjell A.M., Westlye L.T., Amlien I., Espeseth T., Reinvang I., Raz N., Agartz I., Salat D.H., Dale A.M., Walhovd K.B. (2009). High consistency of regional cortical thinning in aging across multiple samples. Cereb. Cortex.

[bb0090] Golomb J.D., Peelle J.E., Addis K.M., Kahana M.J., Wingfield A. (2008). Age differences in temporal and semantic associations in free and serial recall. Mem. Cognit..

[bb0095] Good C.D., Johnsrude I.S., Ashburner J., Henson R.N.A., Friston K.J., Frackowiak R.S.J. (2001). A voxel-based morphometric study of ageing in 465 normal adult human brains. NeuroImage.

[bb0100] Gordon-Salant S., Fitzgibbons P.J. (2001). Sources of age-related recognition difficulty for time-compressed speech. J. Speech Lang. Hear. Res..

[bb0105] Greenwood P.M. (2000). The frontal aging hypothesis evaluated. J. Int. Neuropsychol. Soc..

[bb0110] Grieve S.M., Clark C.R., Williams L.M., Peduto A.J., Gordon E. (2005). Preservation of limbic and paralimbic structures in aging. Hum. Brain Mapp..

[bb0115] Gutchess A.H., Welsh R.C., Hedden T., Bangeert A., Minear M., Liu L.L., Park D.C. (2005). Aging and the neural correlates of successful picture encoding: frontal activations compensate for decreased medial–temporal activity. J. Cogn. Neurosci..

[bb0120] Haug H. (1987). Brain sizes, surfaces, and neuronal sizes of the cortex cerebri: a stereological investigation of man and his variability and a comparison with some mammals (primates, whales, marsupials, insectivores, and one elephant). Am. J. Anat..

[bb0125] Hutton C., De Vita E., Ashburner J., Deichmann R., Turner R. (2008). Voxel-based cortical thickness measurements in MRI. NeuroImage.

[bb0130] Hutton C., Draganski B., Ashburner J., Weiskopf N. (2009). A comparison between voxel-based cortical thickness and voxel-based morphometry in normal aging. NeuroImage.

[bb0135] Im K., Jo H.J., Mangin J.-F., Evans A.C., Kim S.I., Lee J.-M. (2010). Spatial distribution of deep sulcal landmarks and hemispherical asymmetry on the cortical surface. Cereb. Cortex.

[bb0140] Klein A., Andersson J., Ardekani B.A., Ashburner J., Avants B., Chiang M.-C., Christensen G.E., Collins D.L., Gee J., Hellier P., Song J.H., Jenkinson M., Lepage C., Rueckert D., Thompson P., Vercauteren T., Woods R.P., Mann J.J., Parsey R.V. (2009). Evaluation of 14 nonlinear deformation algorithms applied to human brain MRI registration. NeuroImage.

[bb0510] Lemaître H., Crivello F., Grassiot B., Alpérovitch A., Tzourio C., Mazoyer B. (2005). Age- and sex-related effects on the neuroanatomy of healthy elderly. NeuroImage.

[bb0145] Letowski T., Poch N. (1996). Comprehension of time-compressed speech: effects of age and speech complexity. J. Am. Acad. Audiol..

[bb0150] Light L.L., Prull M.W., Kennison R.F. (2000). Divided attention, aging, and priming in exemplar generation and category verification. Mem. Cognit..

[bb0155] Lindenberger U., Baltes P.B. (1994). Sensory functioning and intelligence in old age: a strong connection. Psychol. Aging.

[bb0160] Lohmann G., von Cramon D.Y., Colchester A.C.F. (2008). Deep sulcal landmarks provide an organizing framework for human cortical folding. Cereb. Cortex.

[bb0165] Lustig C., Buckner R.L. (2004). Preserved neural correlates of priming in old age and dementia. Neuron.

[bb0170] Markowitsch H.J., Tulving E. (1994). Cognitive processes and cerebral cortical fundi: findings from positron–emission tomography studies. Proc. Nat. Acad. Sci. U. S. A..

[bb0175] McIntosh A.R., Sekuler A.B., Panpeci C., Rajah M.N., Grady C.L., Sekuler R., Bennett P.J. (1999). Recruitment of unique neural systems to support visual memory in normal aging. Curr. Biol..

[bb0180] Mechelli A., Price C.J., Friston K.J., Ashburner J. (2005). Voxel-based morphometry of the human brain. Methods Appl..

[bb0185] Miller A.K.H., Alston R.L., Corsellis J.A.N. (1980). Variation with age in the volumes of grey and white matter in the cerebral hemispheres of man: measurements with an image analyser. Neuropathol. Appl. Neurobiol..

[bb0190] Naveh-Benjamin M. (2000). Adult age differences in memory performance: tests of an associative deficit hypothesis. J. Exp. Psychol. Learn. Mem. Cogn..

[bb0195] Park D.C., Polk T.A., Park R., Minear M., Savage A., Smith M.R. (2004). Aging reduces neural specialization in ventral visual cortex. Proc. Natl. Acad. Sci..

[bb0200] Peelle J.E., Wingfield A. (2005). Dissociations in perceptual learning revealed by adult age differences in adaptation to time-compressed speech. J. Exp. Psychol. Hum. Percept. Perform..

[bb0205] Peelle J.E., Troiani V., Wingfield A., Grossman M. (2010). Neural processing during older adults' comprehension of spoken sentences: age differences in resource allocation and connectivity. Cereb. Cortex.

[bb0210] Peress N.S., Kane W.C., Aronson S.M. (1973). Central nervous system findings in a tenth decade autopsy population. Prog. Brain Res..

[bb0215] Piao X., Hill R.S., Bodell A., Chang B.S., Basel-Vangaite L., Straussberg R., Dobyns W.B., Qasrawi B., Winter R.M., Innes A.M., Voit T., Ross M.E., Michaud J.L., Déscarie J.-C., Barkovich A.J., Walsh C.A. (2004). G protein-coupled receptor-dependent development of human frontal cortex. Science.

[bb0220] Poldrack R.A., Fletcher P.C., Henson R.N., Worsley K.J., Brett M., Nichols T.E. (2008). Guidelines for reporting an fMRI study. NeuroImage.

[bb0225] Prinz A.A., Bucher D., Marder E. (2004). Similar network activity from disparate circuit parameters. Nat. Neurosci..

[bb0230] Rakic P. (1988). Specification of cerebral cortical areas. Science.

[bb0235] Raz N., Gunning F.M., Head D., Dupuis J.H., McQuain J., Briggs S.D., Loken W.J., Thornton A.E., Acker J.D. (1997). Selective aging of the human cerebral cortex observed in vivo: differential vulnerability of the prefrontal gray matter. Cereb. Cortex.

[bb0240] Raz N., Gunning-Dixon F.M., Head D., Dupuis J.H., Acker J.D. (1998). Neuroanatomical correlates of cognitive aging: evidence from structural magnetic resonance imaging. Neuropsychology.

[bb0245] Raz N., Lindenberger U., Rodrigue K.M., Kennedy K.M., Head D., Williamson A., Dahle C., Gerstorf D., Acker J.D. (2005). Regional brain changes in aging healthy adults: general trends, individual differences and modifiers. Cereb. Cortex.

[bb0250] Resnick S.M., Pham D.L., Kraut M.A., Zonderman A.B., Davatzikos C. (2003). Longitudinal magnetic resonance imaging studies of older adults: a shrinking brain. J. Neurosci..

[bb0255] Rorden C., Brett M. (2000). Stereotaxic display of brain lesions. Behav. Neurol..

[bb0260] Rybash J.M. (1996). Implicit memory and aging: a cognitive neuropsychological perspective. Dev. Neuropsychol..

[bb0265] Salat D.H., Buckner R.L., Snyder A.Z., Greve D.N., Desikan R.S.R., Busa E., Morris J.C., Dale A.M., Fischl B. (2004). Thinning of the cerebral cortex in aging. Cereb. Cortex.

[bb0270] Salthouse T.A. (1996). The processing-speed theory of adult age differences in cognition. Psychol. Rev..

[bb0275] Shafto M.A., Burke D.M., Stamatakis E.A., Tam P.P., Tyler L.K. (2007). On the tip-of-the-tongue: neural correlates of increased word-finding failures in normal aging. J. Cogn. Neurosci..

[bb0280] Sowell E.R., Peterson B.S., Thompson P.M., Welcome S.E., Henkenius A.L., Toga A.W. (2003). Mapping cortical change across the human life span. Nat. Neurosci..

[bb0285] Taki Y., Goto R., Evans A., Zijdenbos A., Neelin P., Lerch J., Sato K., Ono S., Kinomura S., Nakagawa M., Sugiura M., Watanabe J., Kawashima R., Fukuda H. (2003). Voxel-based morphometry of human brain with age and cerebrovascular risk factors. Neurobiol. Aging.

[bb0290] Taki Y., Kinomura S., Sato K., Goto R., Wu K., Kawashima R., Fukuda H. (2011). Correlation between baseline regional gray matter volume and global gray matter volume decline rate. NeuroImage.

[bb0295] Tisserand D.J., Pruessner J.C., Sanz Arigita E.J., van Boxtel M.P.J., Evans A.C., Jolles J., Uylings H.B.M. (2002). Regional frontal cortical volumes decrease differentially in aging: an MRI study to compare volumetric approaches and voxel-based morphometry. NeuroImage.

[bb0300] Tisserand D.J., van Boxtel M.P.J., Pruessner J.C., Hofman P., Evans A.C., Jolles J. (2004). A voxel-based morphometric study to determine individual differences in gray matter density associated with age and cognitive change over time. Cereb. Cortex.

[bb0305] Tun P.A., Wingfield A. (1999). One voice too many: Adult age differences in language processing with different types of distracting sounds. J. Gerontol. B Psychol. Sci. Soc. Sci..

[bb0310] Tun P.A., O'Kane G., Wingfield A. (2002). Distraction by competing speech in young and older adult listeners. Psychol. Aging.

[bb0315] Tyler L.K., Shafto M.A., Randall B., Wright P., Marslen-Wilson W.D., Stamatakis E.A. (2010). Preserving syntactic processing across the adult life span: the modulation of the frontotemporal language system in the context of age-related atrophy. Cereb. Cortex.

[bb0320] Tzourio-Mazoyer N., Landeau B., Papathanassiou D., Crivello F., Etard O., Delcroix N., Mazoyer B., Joliot M. (2002). Automated anatomical labeling of activations in SPM using a macroscopic anatomical parcellation of the MNI MRI single-subject brain. NeuroImage.

[bb0325] Waters G.S., Caplan D. (2001). Age, working memory, and on-line syntactic processing in sentence comprehension. Psychol. Aging.

[bb0330] West R.L. (1996). An application of prefrontal cortex function theory to cognitive aging. Psychol. Bull..

[bb0335] West R.L. (2000). In defense of the frontal lobe hypothesis of cognitive aging. J. Int. Neuropsychopharmacol. Soc..

[bb0340] Wilke M., Holland S.K., Altaye M., Gaser C. (2008). Template-O-Matic: a toolbox for creating customized pediatric templates. NeuroImage.

[bb0350] Wingfield A., Grossman M. (2006). Language and the aging brain: Patterns of neural compensation revealed by functional brain imaging. J. Neurophysiol..

[bb0345] Wingfield A., Lindfield K.C., Kahana M.J. (1998). Adult age differences in the temporal characteristics of category free recall. Psychol. Aging.

