# Supplementary material for: Adjusting for global effects in voxel-based morphometry: Gray matter decline in normal aging
Source: Neuroimage. 2012 Apr 2;60(2):1503–16. doi: 10.1016/j.neuroimage.2011.12.086 (PMC3898996; doi:10.1016/j.neuroimage.2011.12.086)

Supplemental material for:

Peelle JE, Cusack R, Henson RNA. Adjusting for global effects in voxel-based morphometry: Gray matter decline in normal aging. *NeuroImage*.

Fig. S1. Plots of total intracranial volume (TIV) and total tissue class volume, both raw and after TIV has been removed, for male and female participants, calculated based on the “old” segmentation in SPM8 (cf. Fig. 2 in the main paper).

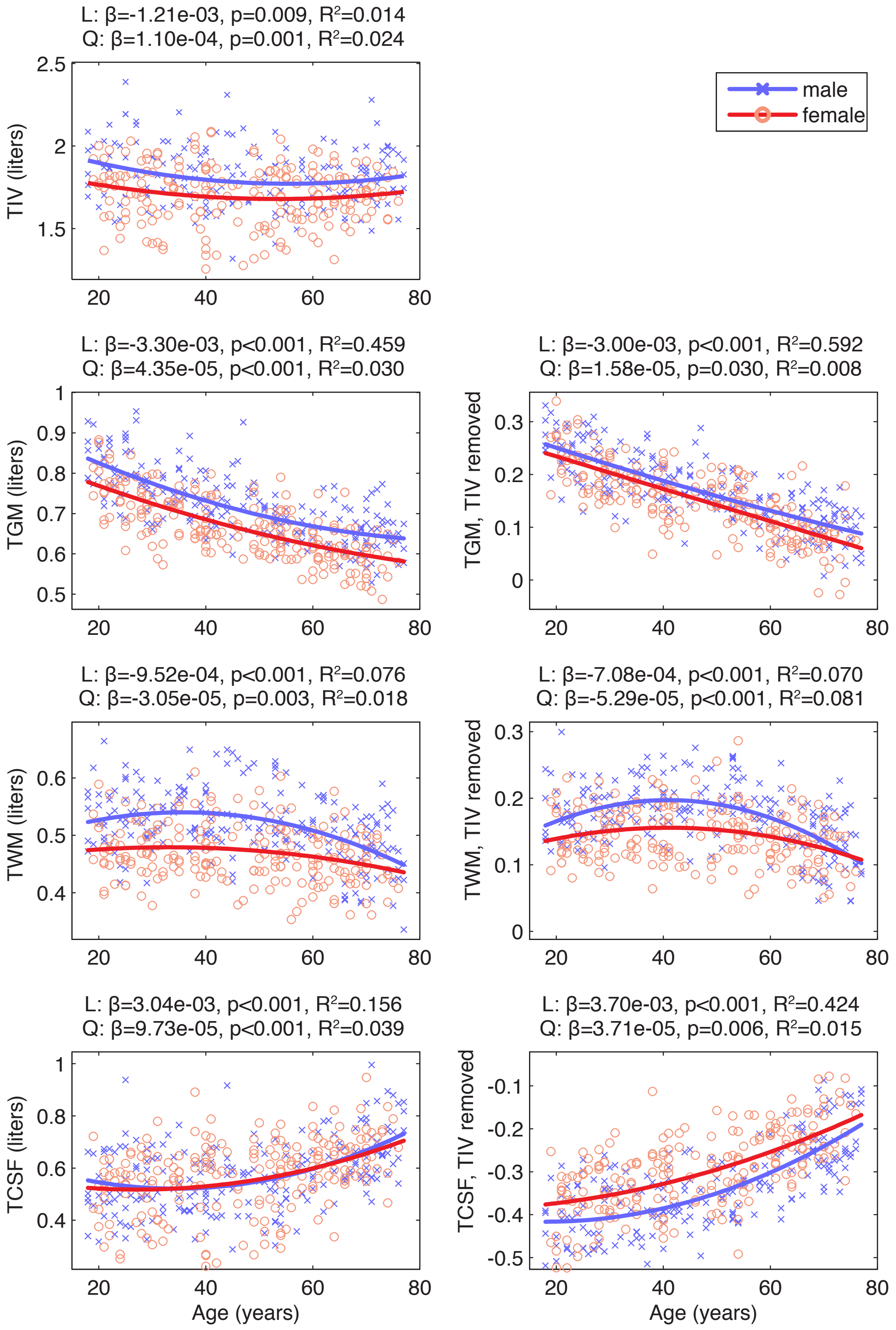

Fig. S2. Correlations between volumes calculated based on two different types of segmentation available in SPM8, split by decile. Note that the slopes of the relationships change with age. The most striking differences, in CSF, are due to the new segmentation including additional tissue classes (and thus less misclassification of other tissue as CSF). TGM = total gray matter, TWM = total white matter, TCSF = total cerebral spinal fluid, TIV = total intracranial volume (TGM+TWM+TCSF).

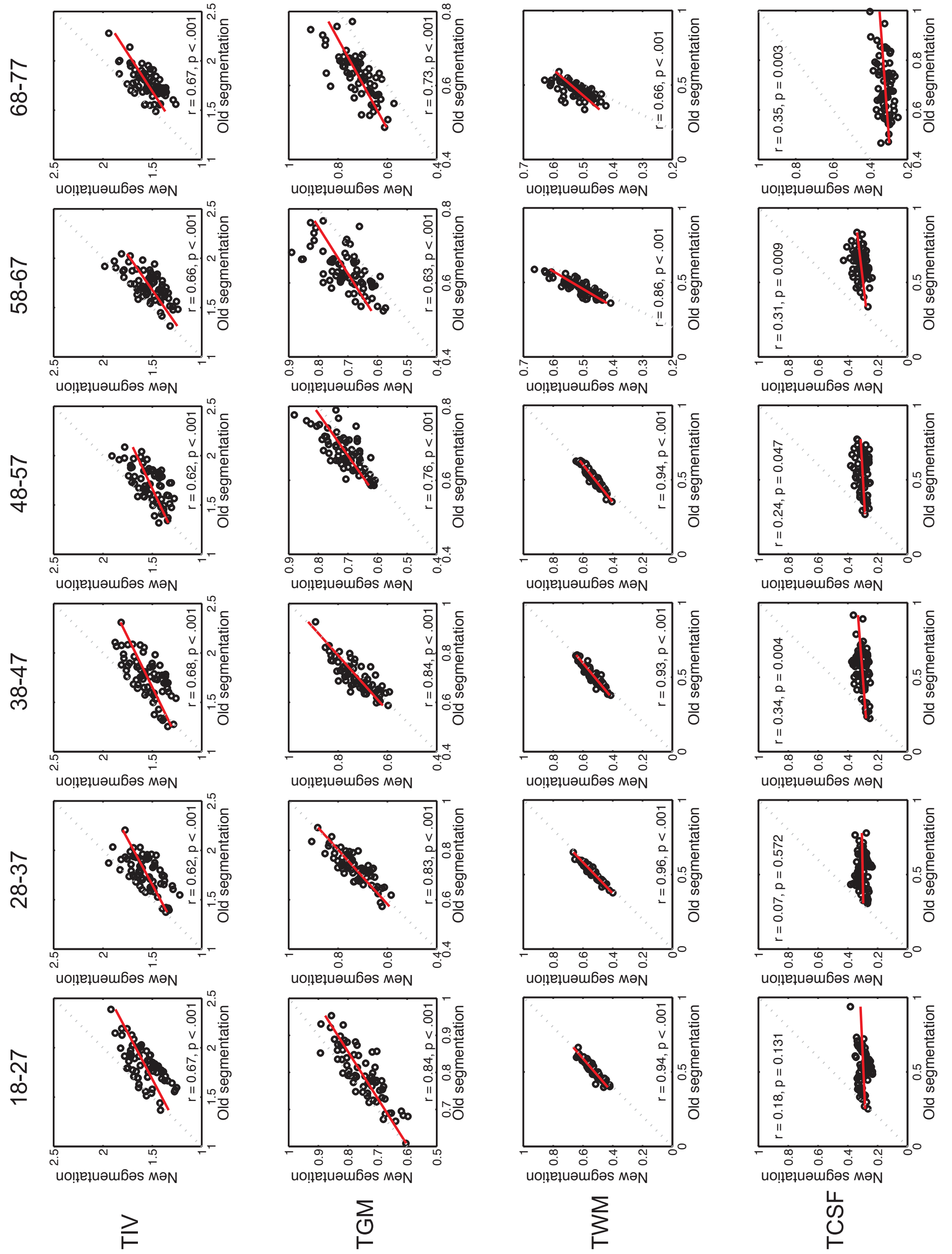

Fig. S3. Correlation among global measures for "old" (a) and "new" (b) segmentation approaches in SPM8. TGM = total gray matter, TWM = total white matter, TCSF = total cerebral spinal fluid, TIV = total intracranial volume (TGM+TWM+TCSF).

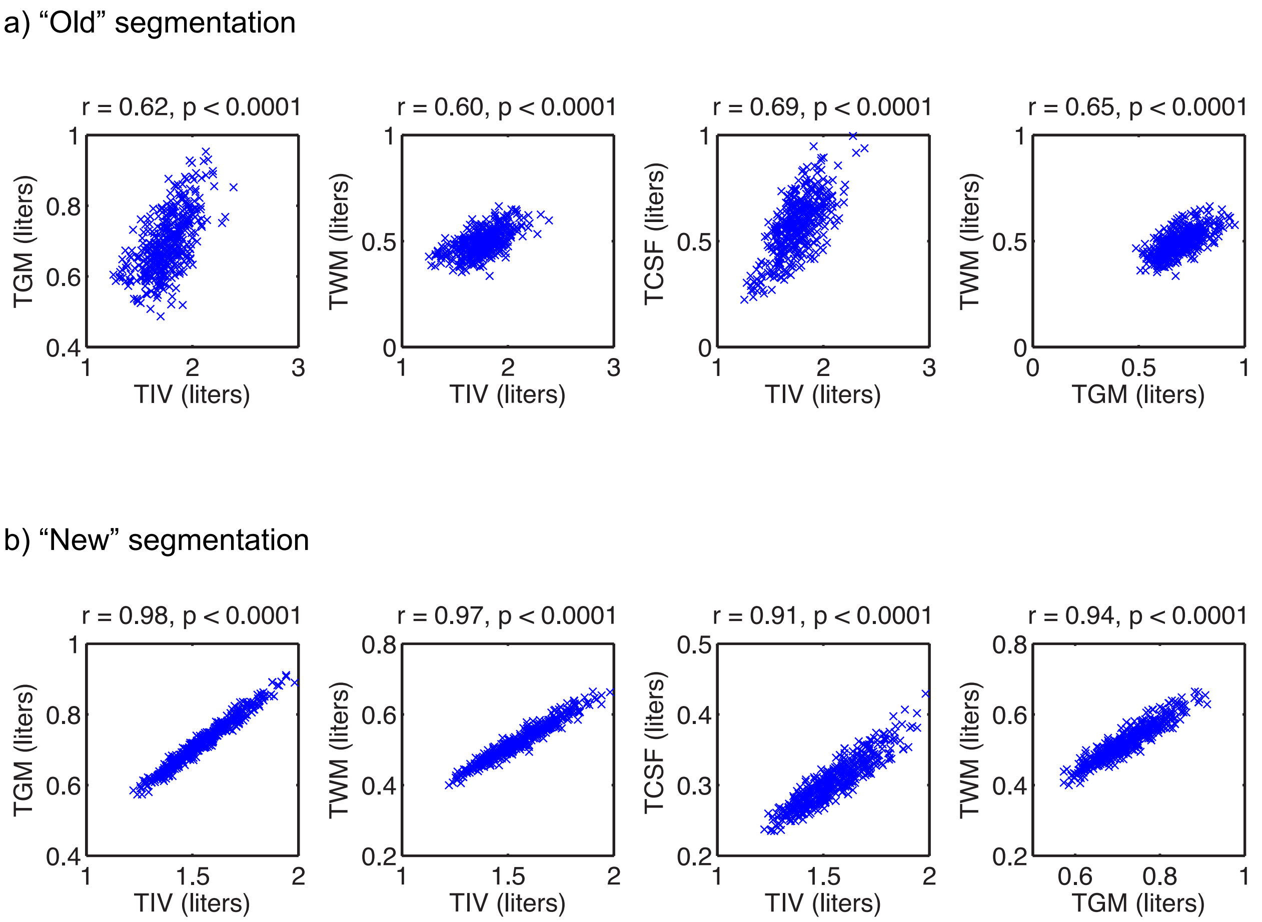

Fig. S4. (a) Linear age-related gray matter declines for three different treatments of total gray matter (TGM), voxelwise $p_{FWE} < .05$, for DARTEL and constrained warp normalization. A region of left insula/inferior frontal gyrus in which statistical significance depends on analysis type is indicated by a white circle. (b) Results for the TGM unaccounted analysis, displayed on the mean unsmoothed gray matter image for each registration. Note the overall increased crispness of the mean of the DARTEL images, as well as the edge effects in the standard registration analysis (white arrows). This may be due to misregistration in the older adults (i.e., normalization not being able to stretch the smaller brains to fit quite as well). In addition, DARTEL results in more focal patterns of atrophy relative to the standard approach.

a)

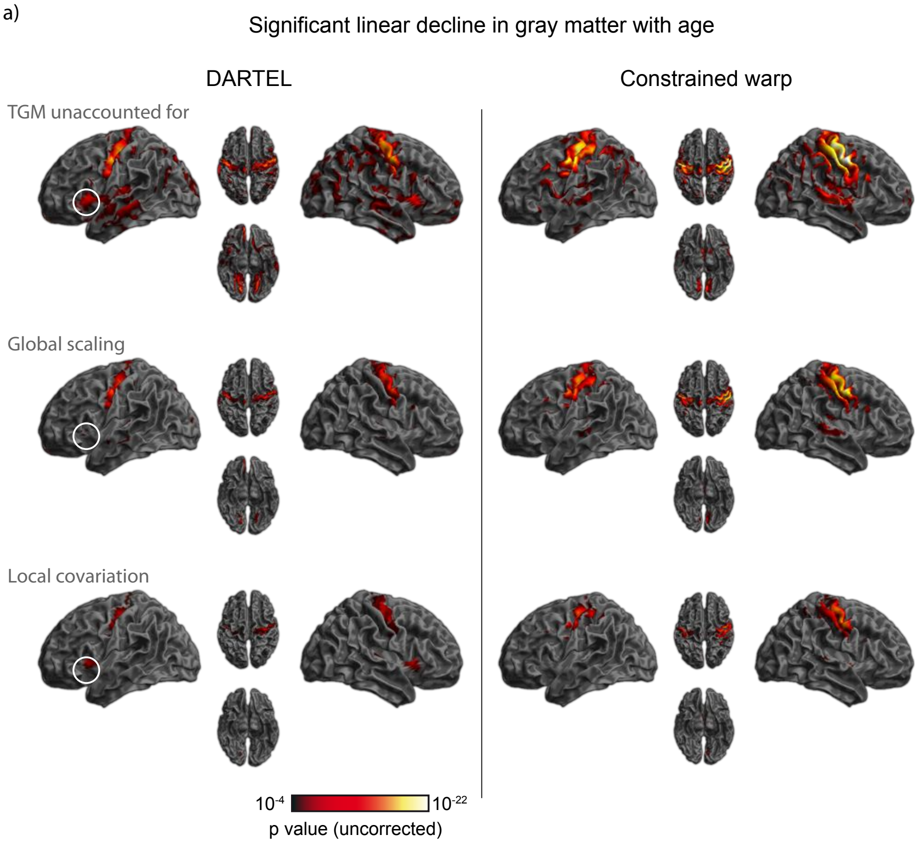

b)

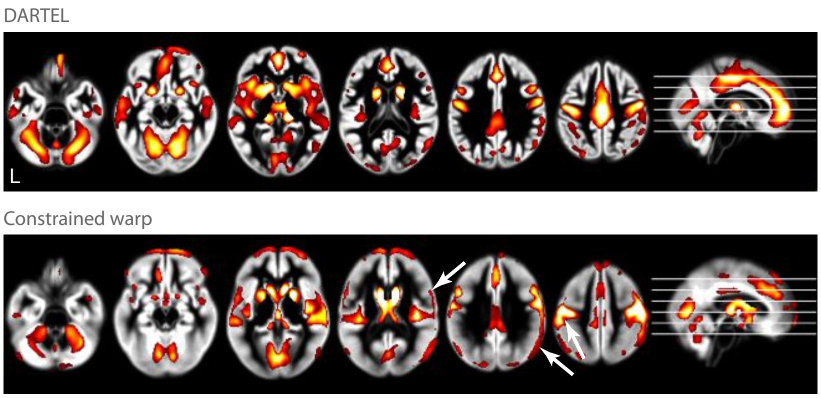

Fig. S5. Quadratic effects of age for three different treatments of total gray matter (TGM): not controlling for TGM, a global scaling approach, and a local ANCOVA approach. The parameter estimate is shown for the average across male and female participants. Positive values on the quadratic term indicate a U shape, negative an inverted U shape. Note that these quadratic effects should be interpreted in the context of the linear effects (Fig. 3).

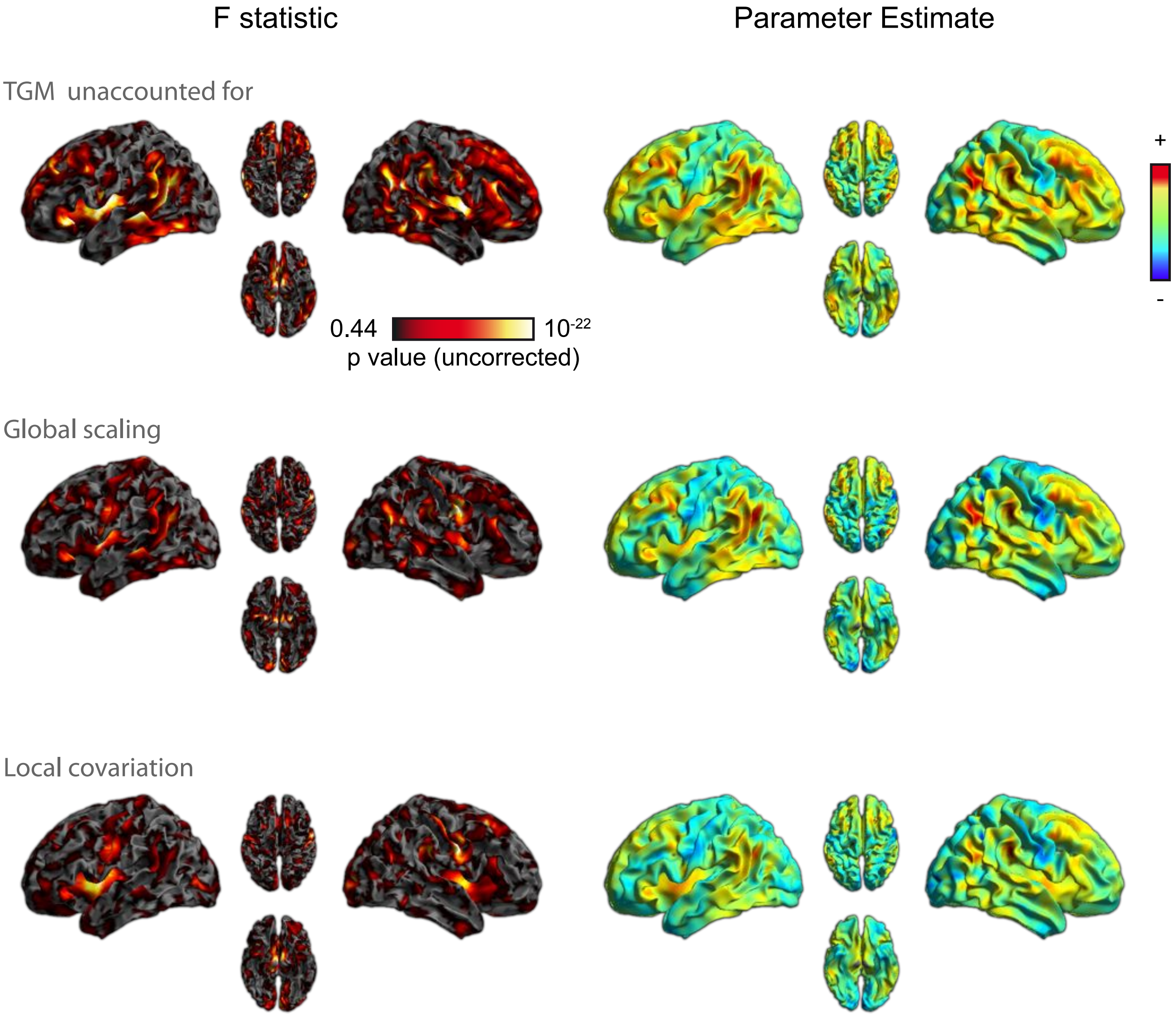

Fig. S6. (a) Mean gray matter volume for unsmoothed DARTEL-registered images displayed on an inflated template brain. (b) Standard deviation of the same images. (c) Residual error form model fit without controlling for TGM. Note that, unsurprisingly, areas of low variability are associated with relatively smaller error, as well as higher t statistics (Fig. 3 in the main paper).

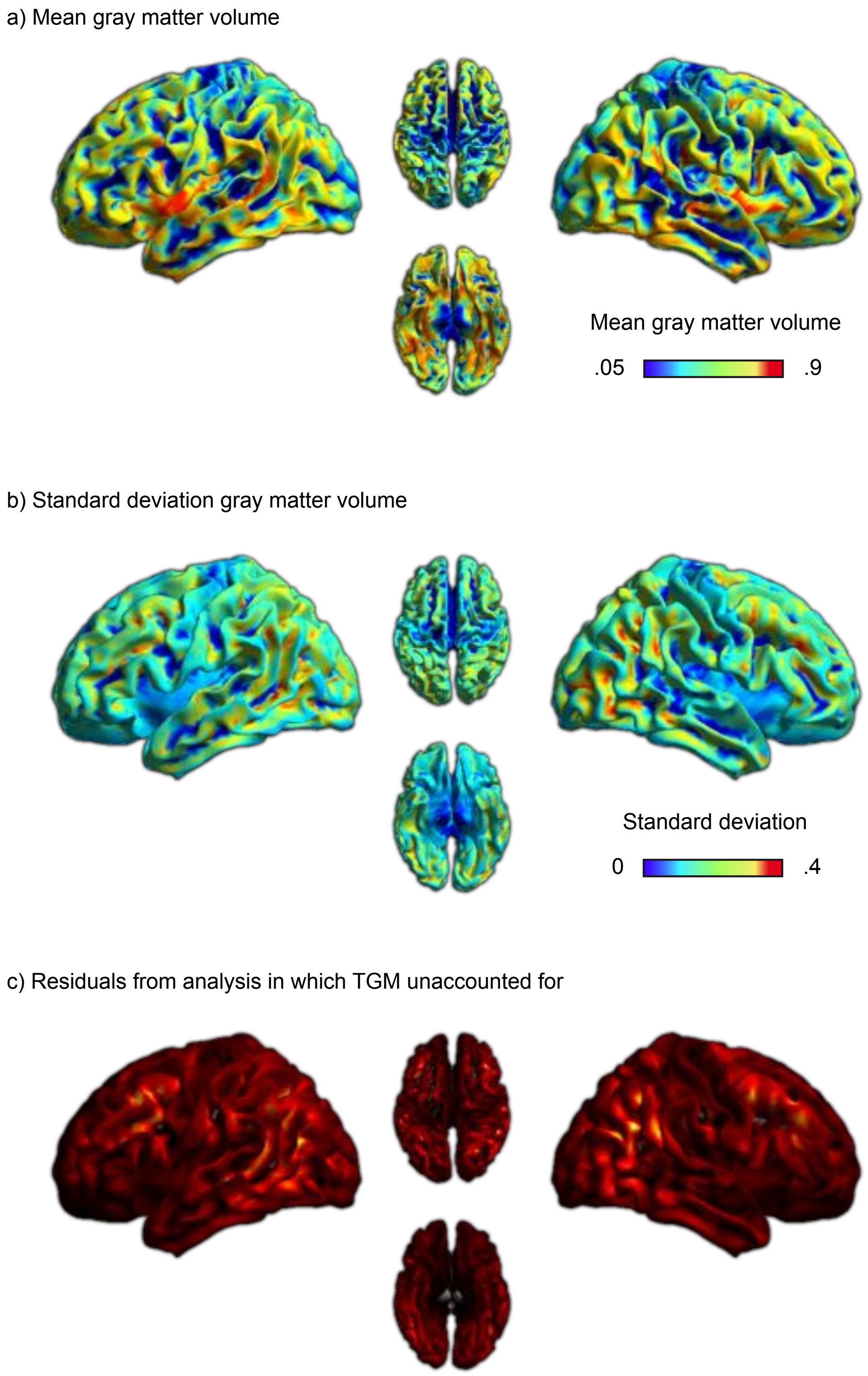

Fig. S7. Analysis of the rate of linear gray matter change in 58 left hemisphere regions of interest (ROIs) (cf. Fig. 5 in the main text). For each of four analysis approaches, ROIs are ranked by the average linear parameter estimate. Highlighted are two pairs of regions whose qualitative relationships change depending on the analysis approach: the amygdala (orange), inferior occipital cortex (green), superior parietal cortex (blue), and pars opercularis of the inferior frontal gyrus (red).

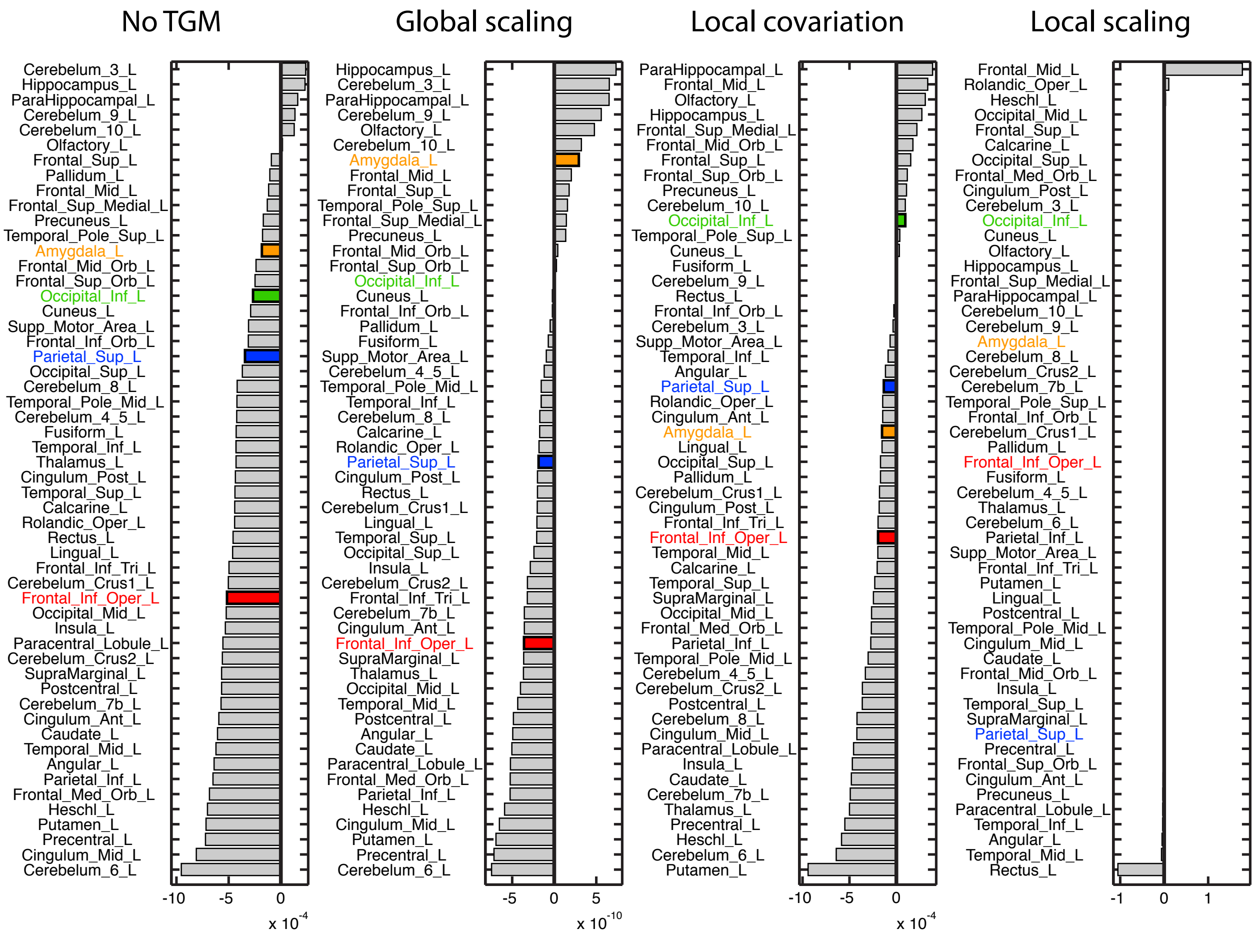

Supplement: Supplementary file 1 — Supplementary materials. [file mmc1.pdf]
